# Distinct landscapes of T-cell immunity and TCR repertoire between sepsis and pre-septic high-risk states

**DOI:** 10.3389/fimmu.2026.1754842

**Published:** 2026-03-03

**Authors:** Yina Ma, Shixiong Shen, Wenjie Zheng, Mingyang Liu, Xiaobao Yang, Yuedan Wang, Xin Zhang

**Affiliations:** 1 Department of Urology Surgery, Beijing Chao-Yang Hospital Affiliated to Capital Medical University, Beijing, China; 2 Department of Anesthesiology, Peking University Third Hospital, Beijing, China; 3 Department of Immunology, School of Basic Medical Sciences, Peking University, Beijing, China

**Keywords:** scRNA-seq, scTCR-seq, sepsis, T lymphocyte, urosepsis

## Abstract

Sepsis remains a leading cause of in-hospital mortality worldwide. In recognition of its substantial morbidity and mortality even with optimal treatment, the World Health Organization has declared sepsis a global health priority. The immunoregulatory mechanisms in sepsis are highly complex, and the immune status during the disease course is closely associated with both short- and long-term patient outcomes, making early recognition and intervention critical for survival. While single-cell RNA sequencing (scRNA-seq) has been widely applied to decipher innate immune responses in sepsis patients, in-depth characterization of T lymphocyte subsets remains relatively limited. Urosepsis is a common complication of urinary tract stones. Although clinical studies have identified several risk factors for urosepsis, the immunological alterations in high-risk individuals are poorly understood. Here, we employed single-cell transcriptomics to investigate T-cell immunological changes in high-risk urosepsis patients and septic patients, complemented by single-cell T cell receptor (TCR) sequencing (scTCR-seq) to profile the peripheral TCR repertoire in these two pathological states. Our analysis revealed that, compared to non-high-risk controls, septic patients exhibited features of T cell exhaustion across multiple subsets, whereas high-risk individuals showed signs of enhanced T cell-mediated adaptive immunity. Notably, we identified a distinct CD4^+^ T cell subset (C10_Tn_IFN) and, through protein-protein interaction analysis, uncovered key protein targets (*IFIT3, RSAD2*) potentially regulating its interferon signaling pathway. Furthermore, we observed significantly reduced TCR diversity accompanied by altered CDR3 sequence characteristics and VJ gene usage frequencies in several CD4^+^ and CD8^+^ T cell subsets from sepsis patients. These findings provide important insights into the relationship between T cell functionality and the severity of infectious inflammation.

## 1 Introduction

Sepsis is a leading cause of in-hospital mortality, accounting for nearly half of all hospital deaths in the U.S (1, 2). Globally, sepsis-related deaths represent 19.7% of all fatalities (2). In 70% of cases deemed unlikely preventable, the primary contributing factor was the severity of illness upon presentation (3). Therefore, early recognition and prevention of sepsis are critical to reducing sepsis-related mortality. Urinary tract diseases represent one of the primary causes of sepsis, with their ranking as an etiology showing a significant upward trend. As a common urological condition, urinary stones can progress to urosepsis after surgery at a rate as high as 13.1% (4). While prognostic factors of sepsis have been extensively studied (5, 6), and predictive models based on clinical data have identified diabetes, cardiovascular disease, urinary tract infections, and infection-related stones as potential high-risk factors for urosepsis in across gender groups (7–10)., the immune mechanisms underlying T-cell-mediated adaptive immunity in the microenvironment of high-risk sepsis populations remain poorly characterized.

Sepsis pathogenesis stems from a dysregulated host immune response to pathogens, involving both innate and adaptive immunity. Immune cells travel systemically via peripheral blood to infection or inflammatory sites, where dysfunction may occur due to altered cell activity, abnormal cell numbers, or disrupted cellular interactions (11–13). While sepsis involves dysregulation across multiple components of the immune system, T cells play a critical role. Upon activation by pathogens or antigen-presenting cells, T cells mount robust immune responses and proliferate extensively to eliminate infections. However, impaired numerical and functional recovery of T cells is not only associated with poor clinical outcomes but may also contribute to long-term immune dysfunction in sepsis survivors (14–16).

In this study, we employed scRNA-seq of peripheral blood mononuclear cells (PBMCs) combined with TCR repertoire analysis to characterize the biological alterations in peripheral blood T cells among sepsis patients. Furthermore, we delineated the immune microenvironment of T cells in a high-risk sepsis cohort, aiming to identify key immunological changes in infected patients. These immunological signatures hold significant clinical value for early identification of high-risk individuals and timely intervention to prevent infection progression to sepsis.

## 2 Materials and methods

### 2.1 Patient cohort and clinical characteristics

This study was approved by the ethics committee of Beijing Chao-Yang Hospital (2024-10-21-4). A total of 10 patients diagnosed with sepsis who met the Sepsis-3 sepsis criteria and was diagnosed within 24h were enrolled in this study. The cohort also included 11 patients with urinary tract stones, among whom 6 were identified as high-risk for urosepsis (HR), while the remaining 5 stone patients without high-risk factors served as the control group (HC); all participants were recruited from Beijing Chao-Yang Hospital. Clinical information was documented for all enrolled participants. Peripheral blood samples from all sepsis patients, 2 HR individuals, and 4 non-high-risk HC subjects were processed for scRNA and scTCR sequencing. A complete list of inclusion and exclusion criteria is presented in **Table 1**, and the detailed clinical characteristics of the sepsis and HR patients included in the scRNA-seq analysis are documented in **Tables 2**, **3**. All participants or their designated surrogates provided informed consent prior to study enrollment.

**Table 1:** Inclusion and Exclusion criteria.

Diagnosis: sepsis (according to Sepsis-3)
Inclusion criteria
Sepsis Documented or suspected infection: lung and urinary tract Organ dysfunction defined as acute change in SOFA score≥2 points
High risk for sepsis
Inclusion criteria
Patients with hydronephrosis secondary to ureteral calculi or other causes, presenting with at least one of the following: 1. Comorbid diabetes mellitus; 2. History of febrile infection within 2 weeks prior to admission; 3. Abnormal laboratory findings: a. White blood cell count (WBC) >20×10^9^/L, b. Platelet count <50×10^9^/L, c. -reactive protein (CRP) >40–50 mg/L; 4. Radiological evidence of perirenal infiltration or septated perirenal fat on CT imaging;
Exclusion criteria
Patients meeting any of the following conditions will be excluded from study participation: 1. Hematologic disorders 2. Active viral infections (including hepatitis B, HIV, or influenza) 3. Chronic infectious diseases (e.g., tuberculosis, syphilis, Lyme disease) 4. History of vaccination within the past month 5. Age <18 years 6. Pregnancy 7. Current use of immunosuppressive agents (including immunoglobulins, corticosteroids, cytotoxic drugs) or immunomodulatory therapies

**Table 2:** Detailed clinical characteristics of HR patients.

Age	77	66
Sex	Female	Female
BMI	24	22.1
Diagnosis	Hydronephrosis(SFU grade III) with ureteral stone	Hydronephrosis(SFU grade III) with urinary tract infection
Diabetes	Yes	No
C-reactive protein (CRP) (mg/L)	14.4	101
Serum albumin (ALB) (g/L)	33.2	28.7
Prealbumin (PA) (mg/L)	96.6	120
Blood lymphocyte count (LYM) ×10^9^/L	0.19	0.13
Creatinine (Cr) (μmol/L)	106	59.7
Procalcitonin (PCT) (ng/mL)	>30	0.5
Blood Culture	Escherichia coli	(-)
Urine WBC (uL)	304	1132
Urine BACT (uL)	1564	64392
Stone history	No	Yes
Malignancy history	No	No
Outcome	Discharged (alive)	Discharged (alive)
SOFA score	1	1

**Table 3:** Clinical characteristics of sepsis patients.

Sample	S02D1	S03D1	S04D1	S05D1	S06D1	S08D1	S10D1	S11D1	S12D1	S15D1
Age	82	92	65	84	70	64	60	85	73	66
Sex	F	M	M	M	F	M	M	F	M	F
Diagnosis	pneumonia	pneumonia	Pneumonia	pneumonia	urosepsis	Urosepsis	urosepsis	urosepsis	urosepsis	urosepsis
Blood Culture	Kleb.p	NA	Negative	Kocuria kristinae	ecoli	Ecoli	NA	ecoli	K. pneumoniae	ecoli
Outcome	Dead	Dead	Alive	alive	alive	Alive	alive	alive	alive	alive

### 2.2 Cell preparation

Fresh blood was separate using Histopaque^®^-1077 (Sigma-Aldrich Catalog No.10771) and Histopaque^®^-1119 (Sigma-Aldrich Catalog No.11191) as instructions. Neutrophils in the cell suspension were removed using MACS^®^- CD15 MicroBeads (No.130-046-601) as instructions. Cell count and viability was estimated using SeekMate Tinitan Fluorescence Cell Counter (SeekGene M002C) with AO/PI reagent after removal erythrocytes (Solarbio R1010) and then dead cells removal was decided to be performed or not (130-090-101). Finally fresh cells were washed twice in the RPMI1640 (Gibco 11875119) and then resuspended at 1×10^6^ cells per ml in RPMI1640 and 2% FBS (Gibco 10100147C).

### 2.3 Single cell RNA-seq library construction and sequencing

Single-cell RNA-seq libraries were prepared using SeekOne^®^ DD Single Cell 5’ library preparation kit (SeekGene Catalog No.K00501). Briefly, appropriate number of cells were mixed with reverse transcription reagent and then loaded to the sample well in SeekOne^®^ DD Chip S3. Subsequently Gel Beads and Partitioning Oil were dispensed into corresponding wells separately in chip. After emulsion droplet generation reverse transcription were performed at 42°Cfor 90 minutes and inactivated at 85°C for 5 minutes. Next, cDNA was purified from broken droplet and amplified in PCR reaction. The amplified cDNA product was fragmented, end repaired, A-tailed and ligated to sequencing adaptor. Finally, the indexed PCR were performed to amplified the DNA which also contained Cell Barcode and Unique Molecular Index. The indexed sequencing libraries were cleanup with VAHTS DNA Clean Beads (Vazyme N411-01), analyzed by Qubit (Thermo Fisher Scientific Q33226) and Bio-Fragment Analyzer (Bioptic Qsep400). The libraries were then sequenced on illumina NovaSeq X Plus with PE150 read length.

### 2.4 Single cell V(D)J-seq library construction and sequencing

Single-cell V(D)J-seq libraries were prepared using SeekOne^®^ DD Single Cell 5’ library preparation kit (SeekGene Catalog No.K00501) and SeekOne^®^ DD Single Cell V(D)J Enrichment Kit (human TCR&BCR, SeekGene Catalog No. K00601&K00701). Briefly, appropriate number of cells were mixed with reverse transcription reagent and then loaded to the sample well in SeekOne^®^ DD Chip S3. Subsequently Gel Beads and Partitioning Oil were dispensed into corresponding wells separately in chip. After emulsion droplet generation reverse transcription were performed at 42°Cfor 90 minutes and inactivated at 85°C for 5 minutes. Next, cDNA was purified from broken droplet and amplified in PCR reaction. Then, the amplified cDNA product from 5′ cDNA was enriched for V(D)J amplification. Next, the V(D)J amplification product was fragmented, end repaired, A-tailed and ligated to sequencing adaptor. Finally, the indexed PCR were performed to amplified the DNA which also contained Cell Barcode and Unique Molecular Index. The indexed sequencing libraries were cleanup with VAHTS DNA Clean Beads (Vazyme N411-01), analyzed by Qubit (Thermo Fisher Scientific Q33226) and Bio-Fragment Analyzer (Bioptic Qsep400). The libraries were then sequenced on illumina NovaSeq X Plus with PE150 read length.

### 2.5 Single cell RNA-seq data processing:

#### 2.5.1 Multiple dataset integration

Raw sequencing data were processed using SeekSoulTools (developed by SEEKGENE, available at: http://seeksoul.seekgene.com/zh/v1.2.2/index.html) for barcode/UMI extraction, reference genome alignment (using an aligner software called **STAR**), and cell barcode filtering. Filtered gene expression matrices were analyzed using SeekSoul Online with Seurat package (4.1.1). Quality control was performed by removing low-quality cells meeting any of the following criteria: (1) <800 or >30,000 unique molecular identifiers (UMIs); (2) <500 or >6,000 detected genes; (3) 10% mitochondrial gene content; (4) 1% erythrocyte gene content. Subsequent analysis included: (1) Batch correction using Harmony (17) to enable cross-condition comparison of three physiological states; (2) Data normalization using the NormalizeData function; (3) Identification of 2,000 highly variable features via FindVariableFeatures; (4) Principal component analysis (PCA) on scaled data (ScaleData function) and dimensionality determination using ElbowPlot; (5) Cell clustering (FindNeighbors and FindClusters functions); (6) Nonlinear dimensional reduction (UMAP implementation). Detailed Seurat analysis protocols are available in the official documentation (https://satijalab.org/seurat/v3.0/pbmc3k_tutorial.html).

#### 2.5.2 Cell-type annotation and cluster marker identification

Cell clusters identified through UMAP projection were annotated by: (1) visualizing canonical marker distribution (FeaturePlot), (2) identifying cluster-specific markers (FindAllMarkers), and (3) validating with SingleR. Doublets (≥2 lineage markers) were excluded.

#### 2.5.3 DEG identification and functional enrichment

Differentially expressed genes (DEGs) were identified using Seurat’s *FindMarkers* (Wilcoxon test, min.pct=0.1, |log_2_FC|≥0.25, Bonferroni correction for false discovery rate (FDR) estimation, FDR<0.05) and analyzed via ORA against Gene Ontology (GO)/KEGG pathways/MSigDB HALLMARK databases. Significant terms were filtered at q-value cutoff < 0.05.

#### 2.5.4 Cell trajectory

We performed single-cell trajectory analysis using Monocle3 (v1.0.0) on preprocessed data (including quality control, normalization, dimensionality reduction, and clustering) to calculate pseudotime trajectories separately for CD4^+^ and CD8^+^ T cell populations.

#### 2.5.5 GEO bulk RNA-seq regression analysis

Bulk RNA-seq data from adult sepsis patients and healthy controls were obtained from the GEO dataset GSE279448. To infer the relative abundance of specific immune cell populations, we calculated gene signature scores for each predefined cell cluster using the Seurat *AddModuleScore* function, based on their top 25 marker genes (and log_2_FC > 0.5). Associations between each cell signature score and sepsis diagnosis were assessed using logistic regression models adjusted for age and sex.

#### 2.5.6 Cell communication

For cell-cell communication analysis, we utilized CellChat v2 (18) with default parameters to analyze intercellular signaling networks, employing gene expression matrices derived from both sepsis patients and sepsis HR groups as input data.

#### 2.5.7 single-cell RNA-seq from septic cohort datasets

Publicly available scRNA-seq data from independent sepsis cohorts were obtained (GSE167363 (19), GSE175453 (20), GSE229173 (21)). We prioritized analysis of samples from patients with sepsis of confirmed bacterial origin (GSM5102902, GSM5102904, GSM5511351, GSM5511353, GSM5511355, GSM5333786, GSM5333789, GSM7156280, GSM7156281, GSM7156282). Additionally, data from healthy donors in dataset GSE217906 were included as controls (GSM6729713, GSM6729714, GSM6729715, GSM8217326, GSM8217327). To ensure that the included transcriptomes primarily originated from viable PBMCs and to avoid wasting a large number of sequencing objects, quality control was performed in batches based on gene expression across different datasets. Specifically, cells from GSE175453 and GSE229173 were excluded if the mitochondrial gene proportion exceeded 10%, the number of genes was less than 200 or greater than 3000, the Unique Molecular Identifier (UMI) count exceeded 15000, or the hemoglobin gene proportion exceeded 2%. The quality control conditions for GSE167363 (excluding mitochondrial genes exceeding 15%) and GSE217906 (excluding UMIs exceeding 10000) were broadly similar. All datasets were reprocessed and analyzed using a unified bioinformatic pipeline (refer to “Multiple dataset integration” above) to ensure comparability using R software (v. 4.4.2) with Seurat package (5.2.1).

### 2.6 TCR analysis

Using the scRepertoire (version 1.8.0) package (22), sample-specific consensus annotation files were consolidated into a list of TCR sequencing results using combineTCR function, and then integrated with the Seurat object using the combineExpression function. Clonotypes were called based on the CDR3 nucleotide sequence. The following diversity and clonality analysis were analyzed following the official tutorial of scRepertoire package. For analysis of *V* and *J* gene usage. Sample clonal diversity (Shannon and chao1 index) was analyzed using R-packet immunarch (version 0.9.0) (23). T cell epitope annotation uses the McPAS-TCR database, and the CDR3 sequence of detected T cells is queried in the database to obtain the corresponding antigen information.

### 2.7 Construction of PPI network

The upregulated genes (log_2_FC ≥ 0.5) from the cell subpopulation were imported into the STRING database (version 12.0; http://string-db.org) to construct a protein-protein interaction (PPI) network (confidence score > 0.9 or 0.7). The resulting network was then imported into Cytoscape (v3.10.3) for further analysis. Highly interconnected molecular modules were identified using the MCODE plugin (v2.0.3) with default parameters (K-core = 2, degree cutoff = 2, max depth = 100, and node score cutoff = 0.2). Hub genes were ranked using two topological algorithms—Maximal Clique Centrality (MCC) and Edge Percolated Component (EPC)—implemented in the cytoHubba plugin (v0.1). Common hub genes were defined as those consistently identified by both methods, ensuring a more biologically robust selection than reliance on node degree alone.

### 2.8 Plasma cytokine detection

Plasma levels of IL-1β, IL-2, IL-4, IL-5, IL-6, IL-8, IL-10, IL-12p70, IL-17, IFN-γ, IFN-α, TNF-α were evaluated by using a 12 Cytokine Assay Kit (Qingdao Raisecare Biotechnology) following the manufacturer’s instructions.

### 2.9 RNA extraction and RT–qPCR

PBMCs were prepared as described above. CD4^+^T cells were purified by negative selection using the EasySep Human CD4^+^T Cell Isolation Kit (STEMCELL Technologies; Cat#17952) according to the manufacturer’s instructions. Total RNA was extracted using TRIzol (Invitrogen) and reverse-transcribed with PrimeScript RT Master Mix (TaKaRa). RT–qPCR was performed using TB Green Premix Ex Taq (TaKaRa) with gene-specific primers (**Table 4**). Expression levels were normalized to β-actin and calculated using the 2^−ΔΔCt method with the control group as the calibrator. qPCR validation was conducted using samples from the same cohort: the 10 sepsis patients and 4 healthy controls (HC) previously analyzed by scRNA−seq. Each sample was run in triplicate for technical replicates to ensure accuracy and reproducibility of results.

**Table 4:** Primer sequences for RT-qPCR.

Primer name	Forward primer (5’ to 3’)	Reverse primer (5’ to 3’)
*IFIT3*	TCAGAAGTCTAGTCACTTGGGG	ACACCTTCGCCCCTTTTCATTTC
*RSAD2*	CAGCGTCAACTATCACTTCACT	AACTCTACTTTGCAGAACCTCAC
*β-actin*	CGCGAGAAGATGACCCAGAT	GGGCATACCCCTCGTAGATG

### 2.10 Statistics

Data are presented as median ± quartiles or as individual values. Box-whisker plots show median, 25th and 75th percentiles and range as vertical lines. Data were compared using MannWhitney Utest or unpaired t test using R software (v.4.4.2). For gene set score analysis, statistical analysis was performed using two-sided Wilcoxon rank-sum tests. In all analyses, a *p* value ≤ 0.05 was considered statistically significant, and all reported *p* values were 2 sided. R packages Seurat (v 5.2.1), ggplot2 (version 3.5.1) were used to generate figures. ∗p < 0.05, ∗∗p < 0.01 and ∗∗∗p < 0.001.

## 3 Results

### 3.1 Single-cell transcriptional profiling of peripheral immune cells

#### 3.1.1 Identification of cell types and their proportional changes

**Figure 1:**
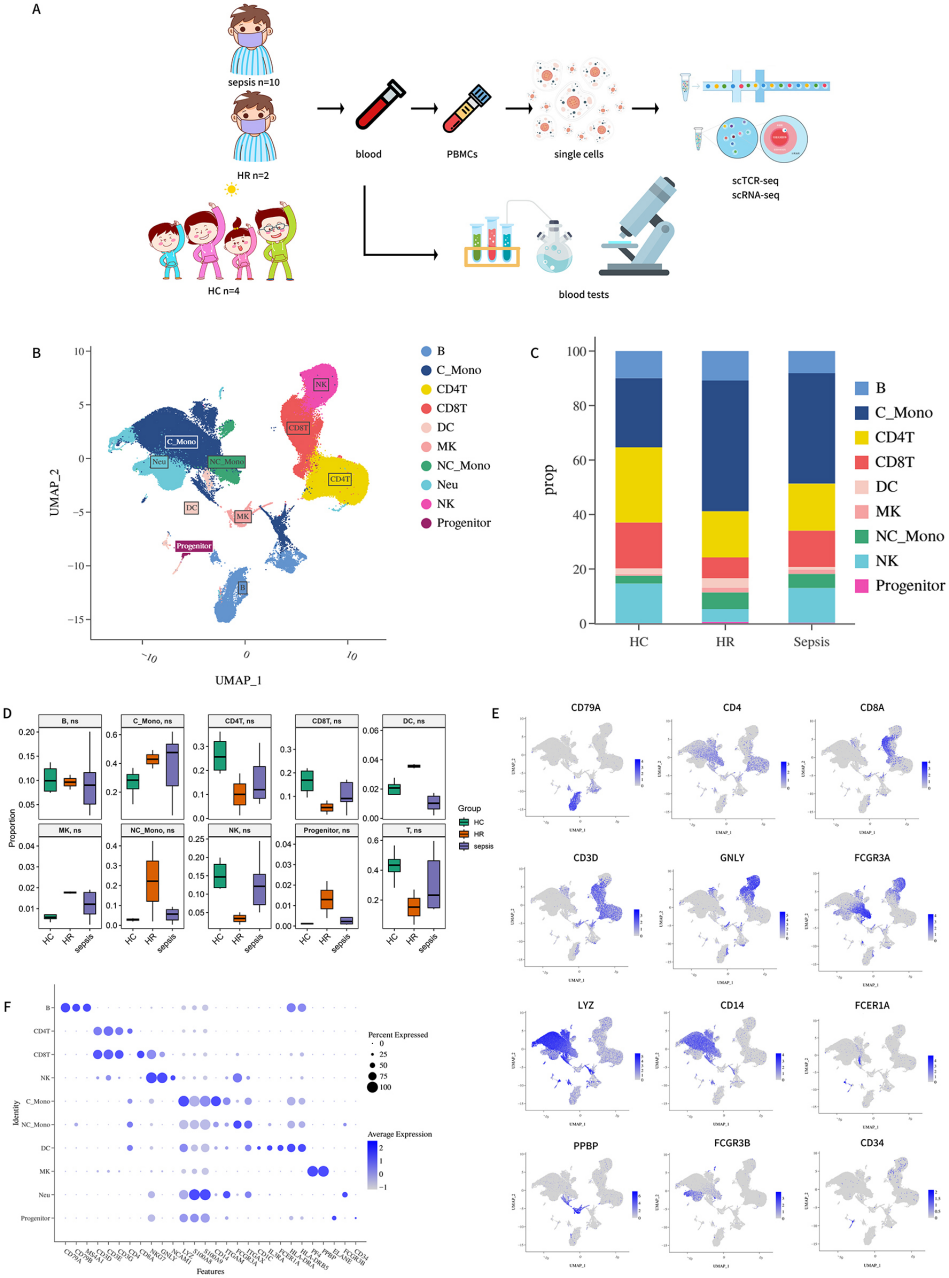
**(A)** Experimental design and schematic workflow of the study. A total of 16 subjects were included: n = 10 with sepsis, n = 4 controls, and n = 2 HR individuals; **(B)** Uniform Manifold Approximation and Projection (UMAP) visualization of 174,636 single cells, colored by annotated cell type; **(C)** Bar plot illustrating the proportional abundance of each cell type within the HC, HR, and sepsis groups; **(D)** Group-wise differences in cell proportions were assessed using the Mann–Whitney U test. p-values < 0.05 were considered statistically significant. Absence of asterisks indicates no significant difference; **(E)** FeaturePlot visualization of marker gene expression across the UMAP embedding. Color intensity corresponds to expression level (log-scaled); **(F)** Dot plots of average expression and percentage of expressed cells of marker genes in each labeled cell type.

To characterize the immunological profiles of sepsis and HR individuals, we performed droplet-based scRNA-seq alongside scTCR-seq on PBMCs isolated from 10 sepsis patients, 2 HR, and 4 non-high-risk HC subjects. Prior to sequencing, CD15^+^ granulocytes were depleted via magnetic sorting to enrich mononuclear cells. Following a unified bioinformatic processing pipeline (see Methods), a total of 174,636 high-quality cells were obtained from all samples. These included 40,839 cells (23.4%) from HC, 24,637 cells (14.1%) from HR, 109,160 cells (62.5%) from sepsis patients. All cells were integrated into a batch-corrected dataset and subjected to principal component analysis. In addition to PBMCs isolation, a comprehensive set of clinical blood tests was conducted to evaluate the subjects’ health status in terms of relevant laboratory indices and clinical scores (**Figure 1A**) (**Tables 5**, **6**).

**Table 5:** Clinical characteristics and cytokine levels in sepsis group vs control group.

Variable	Sepsis (N = 10)	Control (N = 5)	p-value
Age (years)	71.5 (65.2–83.5)	72 (67–73)	0.75900
Temperature (°C)	37.7 (36.5–38.9)	36.4 (36.3–36.4)	0.15600
Respiratory rate (/min)	22 (18.8–24.5)	17.5 (17.2–17.8)	0.15900
Heart rate (/min)	100.5 (88.8–105.5)	76 (73–79)	0.10700
WBC (×10^9^/L)	11.6 (9.5–13.6)	5.8 (4.7–8.1)	0.07580
Neutrophil (×10^9^/L)	9.6 (6.7–12)	3.6 (2.5–5.6)	0.01690
Lymphocyte (×10^9^/L)	0.5 (0.2–1.1)	2 (1.8–2.2)	0.04310
Hb (g/L)	110 (89–139.5)	131 (130–133)	0.19800
PLT (×10^9^/L)	153 (91.2–232.2)	170 (126–254)	0.58200
CRP (mg/L)	126 (108–158)	4.1 (2.6–5.6)	0.04510
PCT (ng/mL)	34.8 (3.4–49.4)	0.1 (0.1–0.1)	0.04090
Albumin (g/L)	31.4 (27.7–33)	38.6 (37.7–41.2)	0.00846
ALT (U/L)	39.8 (18.4–54.9)	16.3 (11.6–23.7)	0.14100
AST (U/L)	35.1 (28–135.8)	15.4 (14.7–16.9)	0.01690
Creatinine (μmol/L)	119.7 (85.5–202.6)	109.8 (63.3–115.1)	0.42600
D-Dimer (mg/L)	2 (1.5–4.5)	0.1 (0.1–0.2)	0.00267
IL-1β	2.58 (0.47–5)	8.47 (2.13–8.76)	0.51900
IL-2	1.33 (0.84–8.47)	2.68 (1.94–2.81)	0.92700
IL-4	0.84 (0.43–1.54)	1.66 (1.42–3.54)	0.26200
IL-5	1.79 (0.79–9)	7.67 (1.68–8.54)	0.92700
IL-6	295.53 (97.08–3269.26)	4.54 (4.47–4.65)	0.00577
IL-8	314.91 (57.83–553.5)	55.4 (17.95–80.17)	0.64800
IL-10	21.59 (8.63–56.97)	1.24 (1.23–1.36)	0.02250
IL-12p70	1.04 (0.7–2.05)	1.36 (1.26–1.76)	0.92700
IL-17	19.73 (4.31–58.16)	4.01 (2.08–4.87)	0.27200
TNF-α	1.09 (0.67–1.51)	2.76 (2.22–3.43)	0.26100
IFN-γ	30.66 (8.69–49.29)	3.54 (1.76–3.93)	0.31100
IFN-α	0.83 (0.58–1.05)	2.78 (1.24–2.97)	0.20000

Continuous variables: median (Q1–Q3). Mann-Whitney U test used for between-group comparisons.

**Table 6:** Clinical characteristics and cytokine levels in high-risk group vs control group.

Variable	High-risk (N = 6)	Control (N = 5)	p-value
Age (years)	71.5 (64.5–77.8)	72 (67–73)	0.8550
Temperature (°C)	37.8 (36.8–38.5)	36.4 (36.3–36.4)	0.1770
Respiratory rate (/min)	20 (18.5–21.5)	17.5 (17.2–17.8)	0.2350
Heart rate (/min)	71.5 (70–95.5)	76 (73–79)	1.0000
WBC (×10^9^/L)	8.8 (6.3–15.4)	5.8 (4.7–8.1)	0.1710
Neutrophil (×10^9^/L)	7.8 (6–14.3)	3.6 (2.5–5.6)	0.0552
Lymphocyte (×10^9^/L)	1 (0.3–2)	2 (1.8–2.2)	0.2350
Hb (g/L)	113 (109.8–118.5)	131 (130–133)	0.0358
PLT (×10^9^/L)	144.5 (109.2–208.2)	170 (126–254)	0.5230
CRP (mg/L)	95.5 (36–141.3)	4.1 (2.6–5.6)	0.0668
PCT (ng/mL)	2.3 (0.6–23.4)	0.1 (0.1–0.1)	0.0651
Albumin (g/L)	31 (27.7–35.6)	38.6 (37.7–41.2)	0.0828
ALT (U/L)	13.4 (12.6–14.5)	16.3 (11.6–23.7)	0.6480
AST (U/L)	19.4 (18.9–21.4)	15.4 (14.7–16.9)	0.2000
Creatinine (μmol/L)	77.7 (53.5–103)	109.8 (63.3–115.1)	0.1710
D-Dimer (mg/L)	1.8 (1.2–2.4)	0.1 (0.1–0.2)	0.0786
IL-1β	2.25 (2.25–2.27)	8.47 (2.13–8.76)	0.9160
IL-2	2.39 (1.11–2.81)	2.68 (1.94–2.81)	0.9170
IL-4	1.58 (0.43–1.64)	1.66 (1.42–3.54)	0.3980
IL-5	2.43 (0.58–3.08)	7.67 (1.68–8.54)	0.6720
IL-6	48 (36.34–685.9)	4.54 (4.47–4.65)	0.0122
IL-8	26.81 (6.87–116.04)	55.4 (17.95–80.17)	0.9170
IL-10	10.81 (1.89–23.71)	1.24 (1.23–1.36)	0.0947
IL-12p70	1.67 (1.59–1.77)	1.36 (1.26–1.76)	0.6760
IL-17	10.36 (7.79–10.67)	4.01 (2.08–4.87)	0.0601
TNF-α	1.95 (0.67–1.96)	2.76 (2.22–3.43)	0.0907
IFN-γ	30.67 (2.71–31.54)	3.54 (1.76–3.93)	0.4030
IFN-α	1.51 (1.25–1.82)	2.78 (1.24–2.97)	0.4630

Continuous variables presented as median (Q1–Q3). Between-group comparisons performed using Mann-Whitney U test.

Based on graph-based clustering of uniform manifold approximation and projection (UMAP), we successfully captured the transcriptomic features of nine major cell types through the expression patterns of canonical gene markers (**Figures 1B, C**). These cell types included: T cells (*CD3D, CD3E, CD3G*; with *CD4* and *CD8A* expression used to broadly distinguish CD4^+^ and CD8^+^ T cell subsets), natural killer cells (NK cells; marker genes *NKG7, GNLY, NCAM1*), B cells (*CD79A, CD79B, MS4A1*), classical monocytes (C_Mono; expressing *LYZ, S100A8, S100A9, CD14, ITGAM*), non-classical monocytes (NC_Mono; markers *FCGR3A, ITGAX*), dendritic cells (DC; marker genes *CD1C, IL3RA, FCER1A*, along with *HLA-DRA* and *HLA-DRB5*), neutrophils (Neu; *ELANE, FCGR3B*), megakaryocytes (MK; *PF4, PPBP*), and progenitor cells (*CD34*) (**Figures 1E, F**). Based on these marker genes, we systematically defined the composition of cellular subpopulations in peripheral blood. Cells co-expressing multiple canonical markers of different lineages were identified as potential doublets/multiplets and were excluded from subsequent analysis.

To uncover differences in cellular composition between the two pathological conditions (sepsis and HR), we calculated the relative percentages of all major cell types in the PBMCs of each individual (**Supplementary Table 2**). We observed a reduction in the median proportion of lymphoid cells (B lymphocytes, T lymphocytes, and NK cells) in sepsis patients compared to controls (24, 25), with a more pronounced decrease in the median percentage of T cells (**Figures 1C, D**). Similar to sepsis patients, HR patients also exhibited a decreased proportion of lymphoid cells relative to controls, with the most notable reduction in T cells. Although these differences in cell proportions did not reach statistical significance between groups, the reduction in T cells was notably substantial in both sepsis and HR patients. Subsequently, we zoomed in on T cell subsets to further dissect their fine-grained subpopulation structures (**Figures 1C, D**).

**Figure 2:**
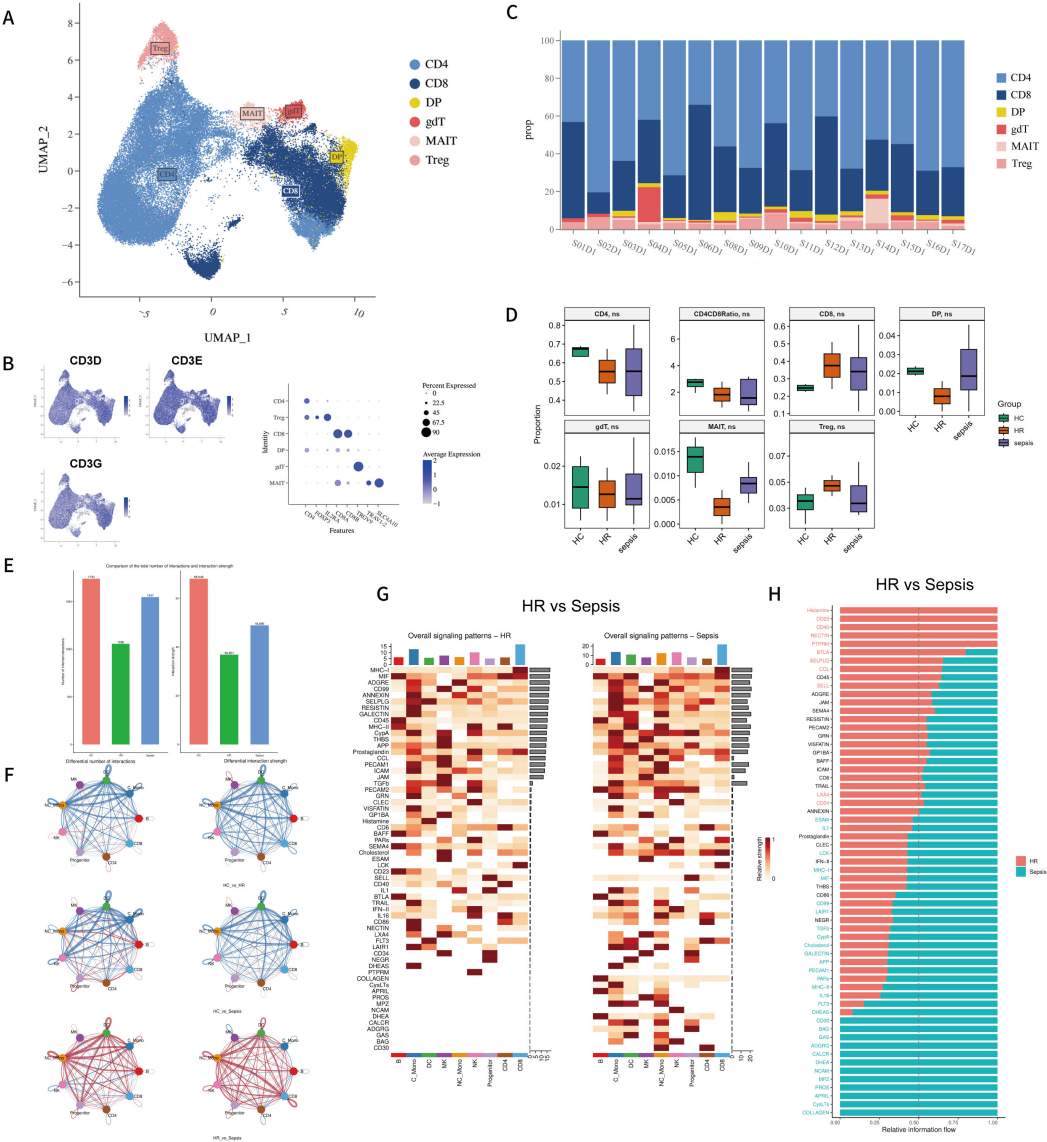
**(A)** UMAP of T cells. Cell types were identified by the marker genes. A total of six T cell subtypes were identified and color-coded; **(B)** FeaturePlot visualization CD3D, CD3E, CD3G gene expression in the UMAP plot of T cell filtered from merged PBMCs. Dot plot of average expression and percentage of expressed cells of selected canonical markers in each labeled cell subtype; **(C)** Bar plot shows the proportional constitution of T cell subtypes in each sample. Colored according to cell type information; **(D)** Boxplots showing the percentage of T cell subtypes across 3 groups (sepsis, HR, HC); **(E)** Bar plot illustrating the total number of ligand–receptor interactions and the overall interaction strength among PBMC subpopulations in each patient group; **(F)** Circle plot comparing the number and communication strength among cell subtypes across different patient groups. Solid circles in different colors represent distinct cell subpopulations. Edge thickness is proportional to the number of ligand–receptor pairs (or communication strength). Edges in red (or blue) indicate increased (or decreased) signaling in the second dataset compared to the first dataset; **(G)** Heatmap showing the overall signaling patterns in HR vs. sepsis patients; **(H)** All major signaling pathways were ranked according to differences in their overall information flow across the inferred networks.

Validation confirmed high expression of *CD3D, CD3E*, and *CD3G* in the aforementioned CD4^+^ and CD8^+^ cell populations; therefore, these two groups were merged into a major T cell cluster. Based on canonical T cell markers, we further identified 6 major T cell subpopulations in the dataset: CD4^+^ T cells (*CD4*), CD8^+^ T cells (*CD8A, CD8B*), γδ T cells (*TRDV2, TRGV9*), MAIT cells (*TRAV1-2, SLC4A10*), Tregs (*FOXP3, IL2RA*), and double-positive T cells (co-expressing *CD4* and *CD8A/CD8B*) (**Figures 2A, B**).

Calculation of intergroup cell proportions revealed that within the T cell compartment of sepsis patients, the median frequencies of CD4^+^ T cells, MAIT cells, and double-positive T cells is inclined toward lower levels compared to controls, consistent with previous studies (26, 27). Among these, the median frequency of CD4^+^ T cells showed the most substantial reduction. In line with established findings (28), we observed a decreased median CD4/CD8 ratio in sepsis patients compared to individuals, suggesting the lymphopenia observed in sepsis and potentially indicating a decline in CD4-mediated specific immune function and an imbalance in our cohort. Conversely, the median proportions of CD8^+^ T cells, Tregs, and γδ T cells were slightly elevated in sepsis patients relative to controls (**Figures 2C, D**).

#### 3.1.2 Global landscape of cell-cell communication

Based on the T cell clustering described above, we performed cell-cell communication analysis on the major cell populations using CellChat. Compared to controls, both the sepsis and HR groups exhibited a reduction in the total number and strength of inferred cellular interactions, indicating overall impairment in cell communication (**Figure 2E**). However, certain interactions showed increased number and intensity, primarily occurring among megakaryocytes (MK), NK cells, B cells, and T cells. These findings suggest that platelets act as critical mediators of inflammatory regulation (29). Notably, septic patients demonstrated a broad surge in both the quantity and intensity of cell-cell communication compared to HR subjects, suggesting extensive involvement of immune cells in interactive immunoregulation (**Figure 2F**).

Both HR and septic groups showed similar alterations in communication patterns related to innate and adaptive immunity, including downregulation of MK, CD48, CD160, TNF, and IL-16 signaling pathways, and upregulation of type II interferon (IFN-II) signaling (**Supplementary Figure 1A**). Subsequently, we observed significant differences in information flow between the two groups: The HR group showed reduced signaling in IL-1, IL-16, and CD30-related pathways, but increases in CD40, BTLA, and CCL signaling. In contrast, the sepsis group exhibited upregulation in communication related to: Cell adhesion and migration (NCAM, PECAM-1, COLLAGEN), Inflammatory activation and inhibitory regulation (APRIL, GAS, CD30, TGF-β) (30–32), and Cell apoptosis (BAG) (**Figures 2G, H**). Furthermore, septic patients exhibit more pronounced inhibitory communication compared to HR individuals, which represents a hallmark of systemic immune dysregulation in sepsis.

### 3.2 Transcriptomic and TCR repertoire alterations in CD4^+^ T cell subsets

#### 3.2.1 CD4^+^ T cells: subpopulation structural and immunological changes

**Figure 3:**
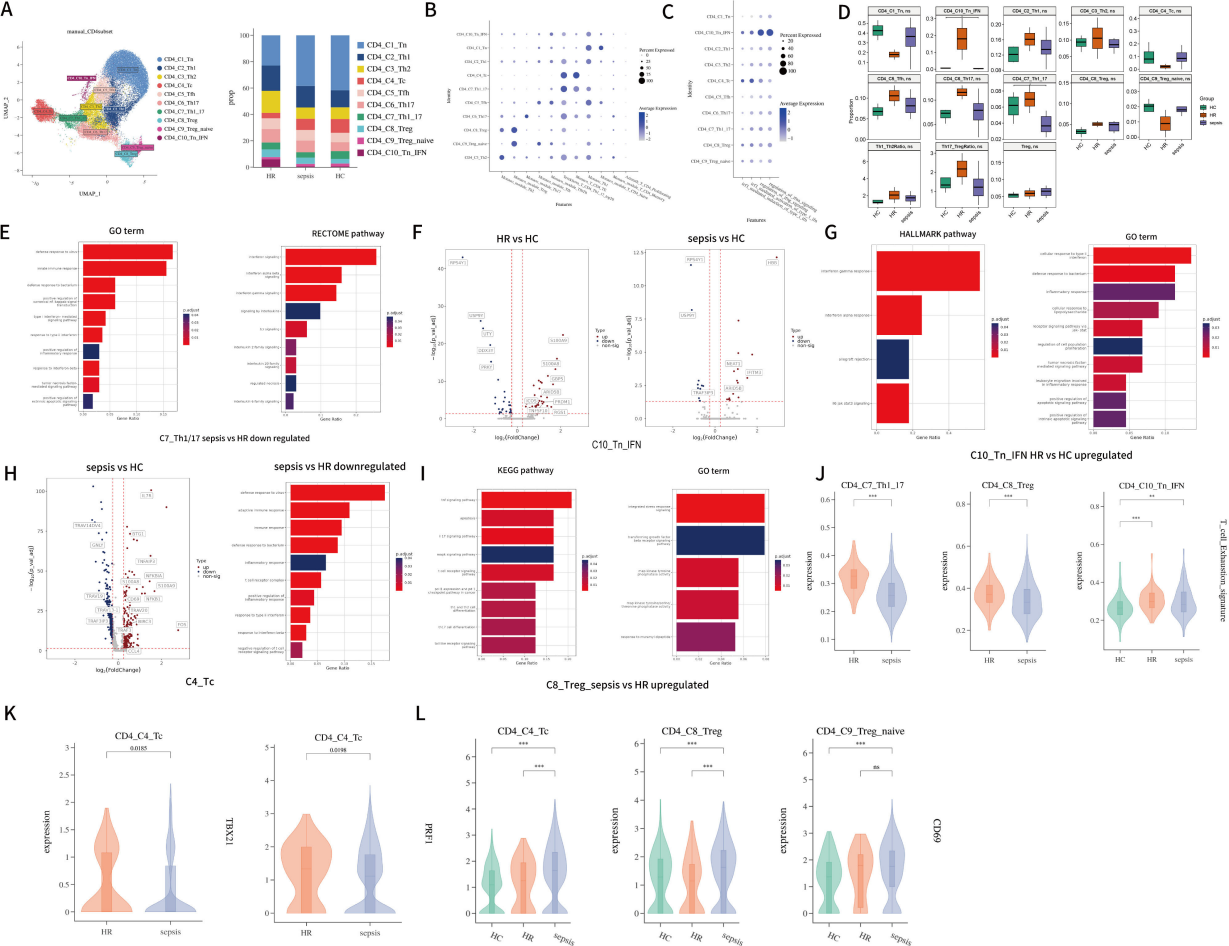
**(A)** UMAP of CD4 T cells. Cell types were identified by the marker genes and subsequent verification of gene set scoring. A total of ten CD4 T cell subtypes were identified and color-coded. Bar plot shows the proportional constitution of CD4 T cell subtypes in each group; **(B)** Gene set scoring plot for publicized genesets (we selected DEG modules published by Monaco et al. (33), the top 20 DEGs of Th1/17 cells summerised by Terekhova et al. (34), the top 15 DEGs of CD4 proliferating cells from Azimuth human PBMC reference (35) for scoring) in each annotated CD4 T subclusters; **(C)** Comparisons of interferon-related pathway (M27240, M26979, M942, M982) module scores across different cell types; **(D)** Group-wise differences in CD4 T subtype proportions were assessed using the Mann–Whitney U test. p-values < 0.05 were considered statistically significant (HR was not included in statistical analysis); **(E)** Functional enrichment analysis (GO terms and REACTOME pathways) of downregulated genes in C7_Th1/17 cells from sepsis patients compared to HR subjects; **(F)** Volcano plot showing the DEGs of C10_Tn_IFN cells in sepsis and HR in comparison to HC; **(G)** Significantly enriched GO terms and Hallmark pathways derived from upregulated DEGs in the C10_Tn_IFN cluster in HR versus HC; **(H)** Volcano plot showing the DEGs of C4_Tc cells in sepsis groups. Representative shared GO terms of downregulated DEGs in sepsis group compared with HR group. **(I)** Significantly enriched GO terms and KEGG pathways derived from upregulated DEGs in the C8_Treg cluster in sepsis versus HR; **(J)** Violin plots visualizing the aggregate expression score for T cell exhaustion (described in PMID: 33293583) (wilcox.test); **(K)** Cytotoxic gene expression is elevated in HR patients compared to septic individuals. (p < 0.05, wilcox.test); **(L)** CD69 expression is elevated in septic patients compared to HR/HC individuals in both cytotoxic CD4 T and regulatory T cells. (∗∗∗p < 0.001, wilcox.test).

The CD4^+^ T cell compartment comprised 32,381 cells, enabling an in-depth investigation of its subpopulation architecture and alterations under different pathophysiological conditions. Based on the expression and distribution of canonical T cell markers, and further validated by gene set scoring against previously published signatures of T cell subsets (as described by Monaco et al. (33) and Terekhova et al. (34)), we identified four major groups encompassing 10 distinct subclusters: naïve cells (cluster 1 and cluster 10), regulatory cells (clusters 8-9), cytotoxic CD4^+^ T cells (cluster 4), and helper cells (clusters 2–3 and clusters 5–7) (**Figure 3A**).

Among these CD4^+^ T cell clusters, four subtypes were annotated using established CD4^+^ subset markers: cluster 1, characterized by high expression of *CCR7, SELL, LEF1*, and *TCF7*, was defined as naïve CD4^+^ T cells (Tn); cluster 4, showing elevated expression of *PRF1, GNLY, NKG7*, and *TBX21*, was identified as cytotoxic CD4^+^ T cells (Tc); and cluster 8, with high expression of *FOXP3* and *IL2RA*, was designated as regulatory T cells (Treg). Helper T cells were preliminarily annotated based on chemokine receptor expression profiles (*CCR4*, *CCR6*, *CXCR5*, and *CXCR3*) (**Supplementary Figure 1B**) and by comparing the highly expressed genes in each cluster with previously published marker genes for helper subsets. This initial annotation was further verified through gene set scoring using the *AddModuleScore* function in Seurat (36), which calculates composite expression scores based on well-curated differentially expressed genes (DEGs) from annotated reference populations (**Figure 3B**) (33–35).

Additionally, we identified a distinct subpopulation (cluster 10) exhibiting high expression of *IFI44L*, *IFIT3*, *CCR7*, and *LEF1*, which was annotated as Tn_IFN. This subset showed enrichment in interferon-related pathways, suggesting that IFN signaling may serve as a potential activator of naïve T cells. As a non-dominant subset of CD4^+^ T cells, Tn_IFN may represent a transient activation state of naïve cells, though this area remains understudied (37). Another subpopulation (cluster 9), characterized by co-expression of *SELL*, *CCR7*, *FOXP3*, and *IL2RA*, was defined as Treg_naïve. Gene set scoring confirmed enrichment of naïve T cell-specific genes in this cluster (**Figures 3B, C**). Top DEGs for each CD4^+^T subset is recorded in **Supplementary Table 1**.

To gain deeper insights into the characteristics of T cell subpopulations, we evaluated the distribution of each cluster across the HC and sepsis (**Supplementary Table 4**). Rank-sum test revealed that the proportions of C7_Th1/17 and C10_Tn_IFN cells were significantly reduced in sepsis patients compared to controls (C7_Th1/17: -2.23%, 95%CI -4.48%~-0.10%, p=0.036; C10_Tn_IFN: -0.32%, 95%CI -0.61%~-0.01%, p=0.036) (**Figure 3D**), consistent with previous findings (38). Although both naïve T cell subsets in this study—C10_Tn_IFN and C1_Tn—exhibited a reduction in median proportion, only the decrease in C10_Tn_IFN reached statistical significance. These findings suggest that pronounced T cell impairment in sepsis are associated with the loss of naïve T cells (39), particularly interferon-primed naïve subsets. The observed reduction in C10_Tn_IFN may be explained by several mechanisms: the high expression of adhesion molecules in this subset indicates a potential for tissue redistribution via migration, or an increased susceptibility to apoptosis within the septic microenvironment.

We observed a decreased Th17/Treg ratio in the enrolled sepsis patients compared to the HC group. Although an increased proportion of C6_Th17 in sepsis patients suggests a the shift toward a Th17-polarized immune response (40, 41), we also found an elevated total proportion of Treg cells (C8_Treg + C9_Treg_naive) in sepsis patients, consistent with earlier reports (42). According to previous literature (43, 44), this finding may suggest that the immune system in these patients is undergoing a cytokine storm followed by inflammatory factor depletion and severe T cell apoptosis. When focusing on changes in Treg cells, the increase in Treg cells could be attributed to reactive expansion of Tregs to suppress excessive inflammatory responses, potentially contributing to sepsis-induced immunosuppression. Between the two Treg subsets, the proportion of C9_Treg_naïve decreased in sepsis patients, while that of C8_Treg increased, possibly reflecting an elevated fraction of effector Tregs and indicating impaired lymphocyte proliferative capacity in sepsis.

Although previous *in vitro* studies have reported significant proliferation of Th17 cells isolated from sepsis patients, we found no significant difference in the baseline proportion of Th17 cells between septic and healthy subjects under unstimulated conditions (40). This observation highlights the limitation of evaluating immune function in sepsis solely based on cellular proportions and underscores the necessity of employing transcriptomic approaches to uncover functional alterations in immune cells (**Figure 3D**).

In HR patients, we observed a propensity for decreased proportion of C1_Tn cells compared to HC, while the proportion of C10_Tn_IFN was elevated relative to HC. This may suggest strong early inflammatory activation, with C10_Tn_IFN cells serving as active responders. Although HR patients demonstrates an upward trend in Treg proportion, the Th17/Treg ratio in this group did not decrease. However, none of these changes reached statistical significance (**Figure 3D**). These findings indicate that HR individuals a trend toward -sepsis-like shifts in T cell subset composition, further investigation using transcriptomic approaches is warranted to explore potential functional alterations in these cells.

#### 3.2.2 Characterization of CD4^+^ T cells in sepsis and high-risk populations

We further investigated the transcriptomic alterations in different CD4^+^ T cell subsets among sepsis and HR patients. Given the significant reduction in the proportions of C7_Th1/17 and C10_Tn_IFN in septic patients, we first focused on functional changes in these subsets. Within the C7_Th1/17 subpopulation, we identified 83 upregulated and 98 downregulated differentially expressed genes (DEGs) in sepsis patients compared to controls. In HR patients, 159 genes were upregulated and 66 were downregulated. The upregulated DEGs in both the sepsis and HR groups were enriched in pathways related to pro-inflammatory effects mediated by cytokines including TNF and IFN, TCR signaling, T cell activation and proliferation, and enhanced immune defense responses (**Supplementary Figure 1C**). These transcriptional signatures suggest that this particular cell subset exhibits heightened transcriptional activity associated with inflammatory activity under both pathological conditions.

Compared to the HR group, sepsis patients exhibited downregulated genes that were enriched in pro-inflammatory pathways such as TNF/IFN signaling and interleukin-mediated responses, TCR-mediated signaling pathways, as well as apoptosis-related pathways (**Figure 3E**). These findings suggest that Th1/17 cells in high−risk individuals display enhanced inflammatory transcriptional activity relative to their septic counterparts. Therefore, the concomitant activation of apoptotic pathways and significant upregulation of exhaustion markers (**Figure 3J**) may represent a compensatory or counter−regulatory response to the heightened inflammatory signaling.

In the C10_Tn_IFN subset of sepsis patients, *IFITM3* and *ARID5B* were identified as significantly upregulated differentially expressed genes (DEGs), while *TRAF3IP3*—a gene critical for maintaining T cell homeostasis—was downregulated (**Figure 3F**) (45). This expression pattern suggests that this cell population is strongly influenced by interferon signaling within the septic microenvironment and may be experiencing inflammatory dysregulation. Previous studies have reported that *ARID5B* plays an important role in promoting the survival of T- and B-lymphocytic leukemia cells, potentially by transcriptionally repressing tumor suppressor genes and enhancing oncogene expression (46). Its upregulation in this context may facilitate T cell survival and proliferation through cell cycle regulation. In HR subjects, the highly expressed 47 DEGs in this subset were predominantly enriched in interferon signaling and multiple pro-inflammatory cytokine pathways (**Figure 3G**). Notably, upregulated expression of *S100A9*, *S100A8*, *PRDM1*, and *ICOS* was observed (**Figure 3F**). The enrichment of these transcriptional pathways, coupled with the upregulation of inflammation−related genes, suggests that IFN signaling may be a key driver of the transcriptional reprogramming observed in this subset. Furthermore, in HR patients, DEGs in this cell population were also enriched in pathways such as “*positive regulation of intrinsic apoptotic signaling pathway*” and “*positive regulation of apoptotic signaling pathway*”, indicating a tendency toward exhaustion (**Figures 3G, J**).

These DEG patterns suggest that both C7_Th1/17 and C10_Tn_IFN may exhibit enrichment of inflammatory response pathways accompanied by features of exhaustion in patients with stones and high-risk factors. When inflammation becomes uncontrolled and progresses to sepsis, the transcriptional activity of these corresponding pathways appears relatively downregulated.

The C4_Tc subset, a cytotoxic CD4^+^ T cell population (47), showed a non-significant increase in proportion under septic conditions compared to controls. We identified 211 upregulated differentially expressed genes (DEGs) in this subset, which were primarily enriched in pathways related to hyperinflammation and immune dysregulation, T cell activation, adhesion, and TCR signaling (**Supplementary Figure 1D**). Interestingly, despite being a cytotoxic subset in sepsis, this population exhibited enrichment in anti-apoptotic pathways (“*negative regulation of apoptotic process*”). Upregulated genes such as *TNFAIP3* (48, 49) and *NFKBIA* (50) suggest that the inflammatory pathways in these cells may be under inhibitory regulation in the septic environment, while elevated expression of *BTG1* may indicate constrained proliferative potential (51). Additionally, high expression of *S100A8*, *CD69*, and *BIRC3* is consistent with an activated state of these cells in sepsis patients (**Figures 3H, L**). HR individuals, however, demonstrates a trend of declining Tc proportion. GO enrichment analysis revealed that the dysregulated pathways in the HR group were similar to those in the sepsis group, yet cytotoxic CD4^+^ T cells (Tc) in HR subjects showed upregulation of inflammatory regulatory pathways relative to the sepsis group (**Supplementary Figure 1D**). Notably, TCR signaling was negatively regulated in HR patients compared to the sepsis group (**Figure 3H**).

HR patients exhibited higher expression of cytolytic granule-related genes compared to septic patients, indicating enhanced transcriptional activity associated with cytotoxicity in Tc cells of the HR group (**Figure 3K**). Overall, compared to septic patients, HR individuals displayed hyperactive yet dysregulated inflammatory responses in Tc cells, characterized by the co-existence of both pro- and anti-inflammatory transcriptional features. Concurrently, TCR-mediated specific immune responses were downregulated. These findings suggest that cytotoxic CD4^+^ T cells exhibit distinct functional tendencies in inflammatory reactions across disease states: they are activated and show elevated expression of cytotoxic genes in the high−risk condition for urosepsis, while TCR-driven adaptive immunity is not yet fully engaged. As in sepsis condition, TCR signaling is upregulated, and so is *CD69* expression (**Figure 3L**), leading to full activation of the adaptive immune response—though this occurs alongside constrained expansion and delayed apoptosis.

A tendency toward increased proportion of regulatory T cells (Tregs) was observed in both HR and septic patients. As Tregs are known to be key mediators of immunosuppression in sepsis (24, 52), we compared their differential gene expression profiles between these two groups and controls. In both patient cohorts, Tregs exhibited significant enrichment of DEGs in pathways related to both immunosuppression and immune activation (**Supplementary Figure 1E**). When comparing septic patients to HR individuals, Tregs in the sepsis group showed widespread upregulation of inflammatory pathways involving cytokine signaling, apoptosis, and antigen-induced activation, alongside features of impaired T cell proliferation and differentiation. Enrichment of the “*PD-L1 expression and PD-1 checkpoint pathway in cancer*” and activation of the “*transforming growth factor beta receptor signaling pathway*” suggest that Tregs in septic patients exert potent immunosuppression through immune checkpoint mechanisms (**Figure 3I**) (52–54). Additionally, upregulation of *CD69* expression indicates full T-cell activation during overt sepsis (**Figure 3L**) (55). These cells also exhibited enhanced survival, homeostasis, and maintenance mechanisms. In contrast, Tregs in HR patients displayed more prominent features of cellular exhaustion (**Figure 3J**). Together, these findings suggest that the immunosuppressive activity of Tregs is closely linked to inflammatory progression and patient outcomes. stronger immunosuppressive effector function appears to be associated with a higher likelihood of progression to sepsis.

Further clinical correlation analyses revealed that the frequencies of specific cellular clusters were significantly associated with the likelihood of sepsis diagnosis (**Table 7**). Notably, an increased frequency of Treg cells showed a positive correlation with sepsis diagnosis (OR = 1.07, 95%CI 1.00-1.13, P = 0.038), whereas a higher frequency of Tc cells was inversely associated with sepsis diagnosis (OR = 0.97, 95%CI 0.94-0.99, P = 0.003). These correlations suggest that the transcriptional mechanisms described above may play an important role in disease progression.

**Table 7:** Univariate and multivariate logistic regression analysis of cell cluster signatures associated with sepsis diagnosis.

Variable	Healthy control (N = 43)	Sepsis (N = 116)	Univariate logistic regression, OR (95%CI)	Multivariate logistic regression, OR (95%CI)
C1_Tn	-23.3 ± 13.9	-55.1 ± 22.6	0.93 (0.91-0.95, p<.001)	0.88 (0.76-1.01, p=.062)
C2_Th1	-59.1 ± 9.6	-88.1 ± 17.5	0.85 (0.81-0.90, p<.001)	0.95 (0.84-1.08, p=.428)
C3_Th2	103.2 ± 35.8	119.3 ± 96.9	1.00 (1.00-1.01, p=.289)	0.98 (0.96-1.01, p=.205)
C4_Tc	340.8 ± 211.1	-25.4 ± 137.3	0.99 (0.98-0.99, p<.001)	0.97 (0.94-0.99, p=.003)
C5_Tfh	0.4 ± 2.5	-1.2 ± 3.3	0.86 (0.77-0.96, p=.007)	2.05 (1.11-3.78, p=.021)
C6_Th17	118.9 ± 70.1	104.3 ± 112.8	1.00 (1.00-1.00, p=.425)	0.89 (0.82-0.97, p=.008)
C7_Th1_17	185.9 ± 151.7	115.4 ± 206.8	1.00 (1.00-1.00, p=.046)	1.05 (1.01-1.09, p=.013)
C8_Treg	-23.1 ± 17.7	-51.8 ± 24.2	0.95 (0.93-0.97, p<.001)	1.07 (1.00-1.13, p=.038)
C9_Treg_naive	3.3 ± 3.5	-1.7 ± 3.7	0.71 (0.63-0.80, p<.001)	0.44 (0.25-0.80, p=.007)
C10_Tn_IFN	-119.2 ± 25.7	-162.6 ± 72.7	0.99 (0.98-1.00, p<.001)	1.00 (0.97-1.03, p=.929)
age	63.2 ± 10.5	63.5 ± 15.0	1.00 (0.98-1.03, p=.900)	1.00 (0.90-1.11, p=.946)
gender	16 (37.2%)	38 (32.8%)		
	27 (62.8%)	78 (67.2%)	1.22 (0.59-2.52, p=.599)	2.09 (0.26-17.04, p=.489)

#### 3.2.3 T cell receptor repertoire profiling of CD4^+^ T cells in sepsis and high-risk populations

##### 3.2.3.1 CDR3 length and TCR V/J gene distribution

**Figure 4:**
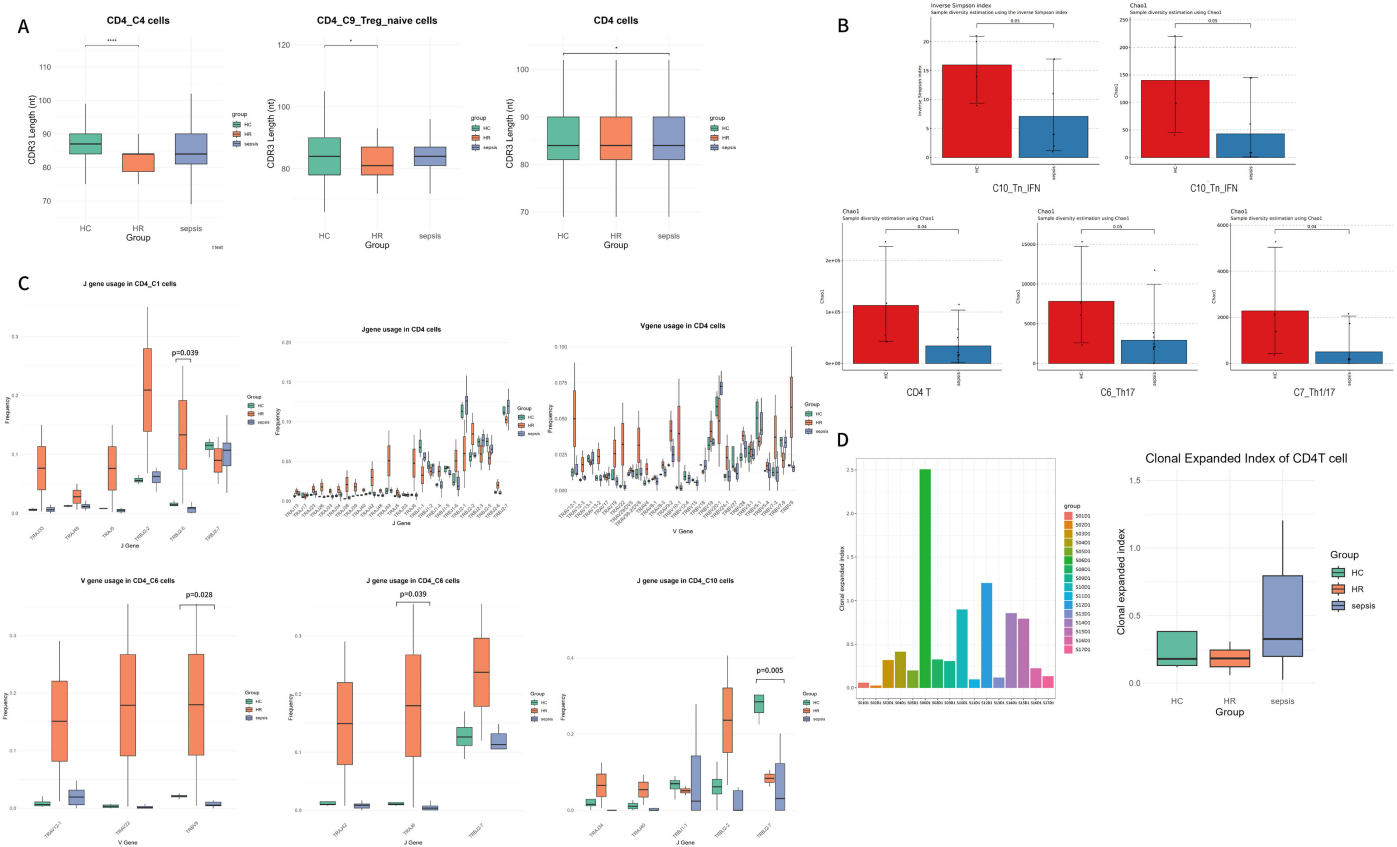
**(A)** nucleotide length distribution of CDR3 in total CD4^+^T cells, C4_Tc, and C9_Treg_naive subsets across HC, HR, and sepsis groups; **(B)** Bar plot showing the TCR repertoire diversity of total CD4^+^T cells and subtypes. Data were analyzed by an unpaired Mann-Whitney U-test. **(C)** Box plot showing the usage frequency of some TCR V and J genes across the three groups in designated subtypes. Statistical significance between groups was determined by unpaired Mann-Whitney U-test; **(D)** Comparison of clonal expansion index across groups and samples. The clonal expansion index is defined as the ratio of the combined frequency of the top 20 clones (each with clone size > 1) to the total frequency of singleton clonotypes (clone size = 1) within each sample. Data were analyzed by an unpaired Mann-Whitney U-test. *p < 0.05, and ****p < 0.0001.

To gain deeper insight into the dynamics of the T cell receptor (TCR) repertoire during sepsis and early immune responses, we reconstructed full-length TCR sequences from sequencing data. We first evaluated the nucleotide length distribution of complementarity-determining region 3 (CDR3) in TCR α and β chains across the overall CD4^+^ T cell population. The results revealed a significant shortening of the average CDR3 length in sepsis patients (M = 84.76, SD = 7.08) compared to HC (M = 84.96, SD = 7.14) (mean difference = 0.20, t = 2.016394, p = 0.044, effect size of Cohen d=0.03) (**Figure 4A**) (56, 57). A trend toward shorter CDR3 sequences was also observed in multiple CD4^+^ T cell subsets from sepsis patients—including C1_Tn, C3_Th2, C4_Tc, C5_Tfh, C6_Th17, C7_Th1/17, C8_Treg, C9_Treg_naive, and C10_Tn_IFN—though these differences did not reach statistical significance. Similarly, HR subjects exhibited shorter average CDR3 lengths in both the overall CD4^+^ T cell pool and specific subsets (C2_Th1, C4_Tc, C5_Tfh, C7_Th1/17, C8_Treg, C9_Treg_naive). Notably, the C4_Tc (mean difference = 3.03, t = 5.66, p <0.001, effect size of Cohen d=0.07) and C9_Treg_naive (mean difference = 2.47, t = 2.24, p = 0.028, effect size of Cohen d=0.03) subsets showed significant shortening compared to HC, suggesting that HR individuals display CDR3 profile alterations resembling those in septic patients (**Figure 4A**). We further compared the TCR VJ gene usage profiles between sepsis and HC groups across CD4^+^ T cell subsets. Key findings in sepsis patients versus HC included: 1) Significantly reduced expression of TRBJ2–6 of C1_Tn (0.78%, 95%CI 0. 16%~1.60%, p=0.039); 2) Markedly decreased usage of TRAJ6 (0.77%, 95%CI 0. 02%~1.66%, p=0.039) and TRBV9 (1.49%, 95%CI 0. 49%~2.11%, p=0.028) in C6_Th17; 3) Significantly lower expression of TRBJ2-7 (22.6%, 95%CI 10. 15%~30.27%, p=0.005) of C10_Tn_IFN (**Figure 4C**). These patterns of biased VJ gene usage partially overlap with previously reported associations in sepsis (58).

##### 3.2.3.2 TCR repertoire diversity and clonality

We next compared the diversity characteristics of the TCR repertoire among septic and HC individuals. Assessment of diversity using the Chao1 index revealed that CD4^+^ T cells from sepsis patients exhibited significantly lower TCR diversity compared to HC (p = 0.04), suggesting a reduction in the overall breadth of the T cell repertoire (**Figure 4B**) (59, 60). Within CD4^+^ T cell subsets from septic patients, decreased diversity—as measured by both the Chao1 and inverse Simpson index (inv.simp)—was broadly observed across multiple subpopulations: C1_Tn, C2_Th1, C3_Th2, C5_Tfh, C6_Th17 (Chao1 p = 0.05), C7_Th1/17 (Chao1 p = 0.04), C8_Treg, C9_Treg_naïve, and C10_Tn_IFN (inverse Simpson p = 0.05, Chao1 p = 0.05) (**Supplementary Figure 1F**). Notably, C6_Th17, C7_Th1/17, and C10_Tn_IFN showed particularly pronounced declines in TCR diversity (**Figure 4B**).

Both the HR and sepsis groups demonstrated clonal expansion within T cell subsets, though the differences did not reach statistical significance when compared with the HC group. (**Figure 4D**).

### 3.3 Transcriptomic and TCR repertoire alterations in CD8^+^ T cell subsets

#### 3.3.1 CD8 T cells: subpopulation structural and immunological changes

**Figure 5:**
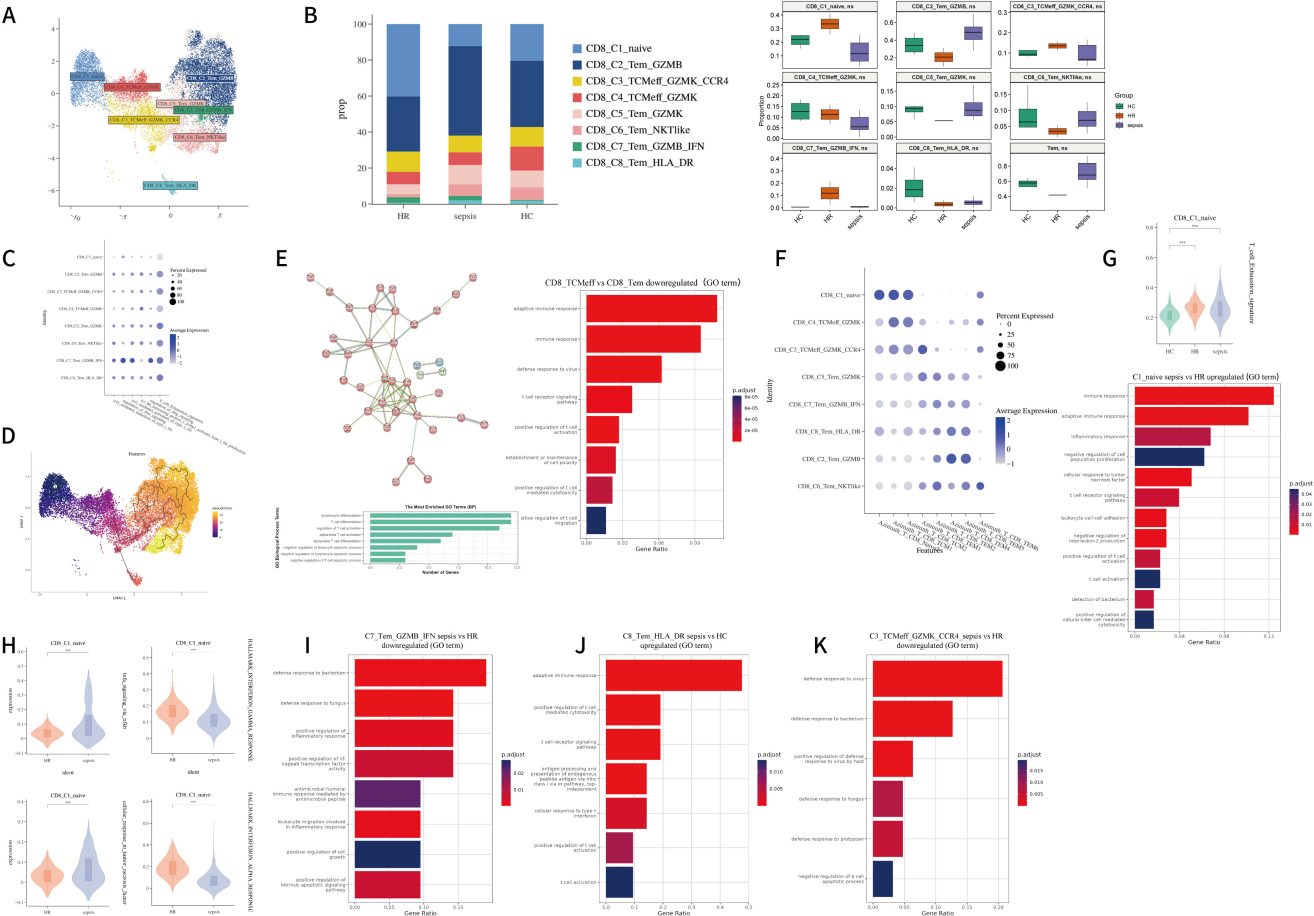
**(A)** UMAP of CD8 T cells. A total of eight CD8 T cell subtypes were identified and color-coded; **(B)** Bar plot shows the proportional constitution of CD8 T cell subtypes in each group. Boxplots showing the percentage of CD8 T cell subtypes across 3 groups; **(C)** Comparisons of interferon-related pathway module scores across different cell types; **(D)** UMAP plot of the pseudotime trajectory of CD8 ^+^ T cells; **(E)** Protein interaction network constructed based on 94 DGEs. GOBP terms of downregulated genes in Tcm_eff cells compared to the Tem cells. **(F)** Gene set scoring of CD8^+^ T cell subsets using signature genes derived from Hao et al. (35) (top 15 upregulated genes per subset were used as reference signatures); **(G)** Violin plots visualizing the aggregate expression score for T cell exhaustion. Significantly enriched GO terms derived from upregulated DEGs in the CD8_C1_naïve cluster in septic individuals versus HR; **(H)** Violin plots depict aggregate expression scores for IFN-related (Hallmark_Interferon_Alpha_Response, M5911; Hallmark_Interferon_Gamma_Response M5913) and TNF-related (tumor necrosis factor-mediated signaling pathway, GO:0071356; tnfa_signaling_via_nfkb, M5890) gene sets. IFN pathways are upregulated in HR subjects, while TNF pathways dominate in sepsis (p < 0.001, Wilcoxon test); **(I)** Functional enrichment analysis (GO terms) of downregulated genes in C7_Tem_GZMB_IFN cells from sepsis patients compared to HR subjects; **(J)** Significantly enriched GO terms in the C8_Tem_HLA_DR cluster in septic individuals versus HC; **(K)** Functional enrichment analysis (GO terms) of downregulated genes in C3_TCMeff_GZMK_CCR4 cells from sepsis patients compared to HR subjects. ***p < 0.001.

The CD8^+^ T cell compartment comprised 14,819 cells. Based on the expression and distribution of canonical CD8^+^ T cell markers and validated by gene set scoring against the T cell subset reference in the Azimuth human PBMC dataset (35), we identified three major groups encompassing eight subclusters: naïve cells (cluster 1), effector memory T cells (Tem, cluster 2, cluster 5-8), and central-effector memory T cells (cluster 3-4) (**Figures 5A, F**). The population designated as central-effector memory T cells (Tcm_eff) was annotated based on its weak expression of naïve T cell markers (*CCR7, SELL*, and *TCF7*), coupled with elevated *IL7R* expression—consistent with central memory T cell (Tcm) characteristics—and concurrent expression of the cytotoxic gene *GZMK*, a feature typical of effector memory T cells (Tem). Furthermore, pseudotemporal trajectory analysis using Monocle3 indicated that this cluster occupies an intermediate state between naïve and effector memory T cells (**Figure 5D**). Genes with higher expression in Tcm_eff compared to the Tem subset (log_2_FC > 0.5, 94 genes in total) were subjected to PPI analysis using STRING (confidence score > 0.7). This identified 39 proteins with interactions, which were grouped into 3 clusters. Among these, the 35 genes in Cluster 1 were significantly enriched in pathways related to negative regulation of T cell apoptosis and T cell activation. Conversely, the 408 downregulated genes were enriched in immune and inflammatory responses, TCR signaling activation, and T cell cytotoxicity, suggesting that this cell population displays a transcriptional downregulation of inflammatory pathways relative to the Tem subset. (**Figure 5E**). We therefore defined this population as Tcm_eff, likely representing a transitional CD8^+^ T cell subset undergoing activation from Tcm to Tem.

Among the three major groups within the CD8^+^ T cell clusters, cell populations were annotated based on canonical CD8^+^ subset markers: Cluster 1, exhibiting high expression of *CCR7*, *SELL*, *LEF1*, and *TCF7*, was defined as naïve CD8^+^ T cells (Tn). Cluster 2, characterized by high expression of *GZMB*, *ZNF683*, *PRF1*, and *GNLY*, was designated as effector memory CD8^+^ T cells_GZMB (Tem_GZMB) (**Supplementary Figure 2A**). A distinct population (Cluster 7) displayed a gene expression profile similar to C2_Tem_GZMB but was marked by high expression of interferon-stimulated genes including *IFI44L*, *IFIT2*, *IFIT3*, and *IFI6*. Gene set enrichment analysis confirmed significant enrichment of interferon-related pathways in this cluster (**Figure 5C**). Consequently, this subset was annotated as Tem_GZMB_IFN. Notably, this population also exhibited elevated expression of exhaustion markers, indicating pronounced functional exhaustion. Cluster 5, showing high expression of *GZMK*, *PRF1*, and *GZMA*, was defined as effector memory CD8^+^ T cells_GZMK (Tem_GZMK). Cluster 6, which expressed cytotoxic genes typical of CD8^+^ T cells along with *NCAM1*, was annotated as natural killer T cell-like (NKT-like). Although Cluster 8 also expressed cytotoxic genes and could broadly be classified as an effector CD8^+^ T population, its most distinctive feature was high *HLA-DRB5* expression. We therefore termed this subset Tem_HLA_DR. Within the identified Tcm_eff population, which exhibits central memory T cell (Tcm) characteristics, we further annotated subclusters based on the expression patterns of chemokine receptors, consistent with previous reports on Tcm biology. The subpopulation with high *CCR4* expression (Cluster 3) was named Tcm_eff_GZMK_CCR4, while the subpopulation with low *CCR4* expression (Cluster 5) was designated Tcm_eff_GZMK. Top DEGs for each CD8^+^T subset is recorded in **Supplementary Table 1**.

We evaluated the distribution of each CD8^+^ T cell subset across the across the sepsis and HC groups (**Supplementary Table 5**) and observed a non-significant trend toward a reduced proportion of CD8^+^ naïve T cells in sepsis patients (**Figure 5B**). Nonetheless, this observed reduction in naïve CD8^+^ T cells among sepsis patients aligns with findings from previous studies (61, 62). Compared to controls, sepsis patients exhibit a tendency toward an increased overall proportion of circulating Tem CD8^+^ T cells (63). Subcluster analysis revealed that most effector memory subsets were elevated in sepsis, with the exception of C8_Tem_HLA_DR, which showed decreased proportions. Additionally, sepsis patients demonstrated a reduced percentage of Tcm_eff cells. Interestingly, we found that the proportion of C3_Tcm_eff_GZMK_CCR4 was modestly decreased in the sepsis group, which may result from downregulation of CCR4 following full activation and effector differentiation of these cells (64).

#### 3.3.2 Characterization of CD8^+^ T cells in sepsis and high-risk populations

We further investigated the transcriptomic alterations in CD8^+^ T cell subsets across patients with sepsis and those at high risk. Our analysis revealed that the C1_naïve subpopulation in both groups exhibited enrichment inflammatory activation pathways, including TNF effector signaling, interferon production, pattern recognition receptor (PRR) activation, and cytokine secretion pathways (**Supplementary Figure 2B**). Additionally, both groups showed enrichment in antigen recognition pathways, including the T cell receptor (TCR) signaling, as well as recognition and responsiveness to bacterial components such as lipopolysaccharide (*response to lipopolysaccharide*). Notably, compared to the sepsis group, the upregulated enrichment pathways in HR subjects demonstrated a tendency toward cell differentiation polarization (*positive regulation of cell differentiation*) and anti-apoptotic mechanisms (*negative regulation of apoptotic process*) within the C1_naïve subset. In contrast, septic patients exhibited signs of immunosuppression and exhaustion (*negative regulation of T cell receptor signaling pathway, PD-1 signaling*) alongside increased programmed cell death (**Supplementary Figure 2B**). Furthermore, in sepsis patients, the C1_naïve subpopulation showed extensive interaction signatures with various circulating non-lymphoid components—such as cells neutrophils, and NK cells—suggesting a broad modulatory influence on blood cell populations.

When directly comparing septic and HR patients, we found that DEGs in the sepsis group were enriched in pathways related to more pronounced immune activation, bacterial recognition, cytotoxic responses, and TCR signaling. However, concurrent enrichment in T cell immunity pathway inhibition (*negative regulation of IL-2 production*), proliferation suppression, and exhaustion markers indicates that patients with overt sepsis experience a cytokine storm that amplifies T cell immunity while also driving increased apoptosis of naïve cells—helping to explain the observed reduction in this population (**Figure 5G**). An intriguing finding was that C1_naïve cells in HR individuals were predominantly enriched in type I/II interferon pathways, whereas in septic patients, the profile shifted toward bacterial recognition and response to inflammatory cytokines such as TNF (**Figure 5H**). This suggests distinct cytokine drivers at different disease backgrounds.

The data suggest a pattern of increased proportion for the C7_Tem_GZMB_IFN subset in both groups, yet its transcriptional characteristics differed markedly between these cohorts. Comparative analysis of differentially enriched pathways between septic and HR patients revealed a broad downregulation of immune-response pathways from sepsis patients, including: immune defense (*defense response to bacterium*, *antimicrobial humoral immune response mediated by antimicrobial peptide*),inflammatory reactivity (*positive regulation of inflammatory response*), migratory capacity (*leukocyte migration involved in inflammatory response*), Concurrent apoptosis (*positive regulation of intrinsic apoptotic signaling pathway*) (**Figure 5I**). These findings suggest that although the C7_Tem_GZMB_IFN subset is active in HR group, its immune functions become broadly suppressed in sepsis, and that interferon signaling may serve as an early activator of CD8^+^ effector memory T cells in this context. Collectively, these results highlight the potential of functional changes in this T cell subset to serve as an early warning indicator for sepsis progression.

The C8_Tem_HLA_DR subset, representing a late-stage activated T cell population, exhibited distinct transcriptional profiles in septic patients compared to controls. Sepsis group showed 24 upregulated genes in this population relative to HC. These genes were enriched in pathways related to interferon activation, T cell activation and cytotoxicity, and antigen presentation (**Figure 5J**).

The C3_Tcm_eff_GZMK_CCR4 subset showed enrichment of upregulated pathways in the HR group, including anti-pathogen responses, interferon-related inflammatory responses, cell migration and tissue homing, as well as cell survival and homeostasis maintenance. These findings suggest that this subpopulation is immunologically activated yet remains capable of maintaining homeostasis in HR patients, potentially serving a key role in sustaining immune memory. In contrast, its proportion was numerically lower in the sepsis group. Compared to controls, septic patients showed upregulation of pathways related to pro-inflammatory and inhibitory signaling, suppression of T cell activation and differentiation, and activation of multiple cell death pathways (**Supplementary Figure 2B**).

Overall, relative to HR subjects, the C3 population in septic patients displayed enrichment of pathways regulated by multiple cytokines (e.g., IFN, TNF, TGF-β), more complex inflammatory regulation, but reduced pathogen-killing capacity and increased apoptosis (**Figure 5K**), indicating a state of inflammatory dysregulation.

#### 3.3.3 T Cell receptor repertoire profiling of CD8^+^ T cells in sepsis and high-risk populations

##### 3.3.3.1 CDR3 length and TCR V/J gene distribution

**Figure 6:**
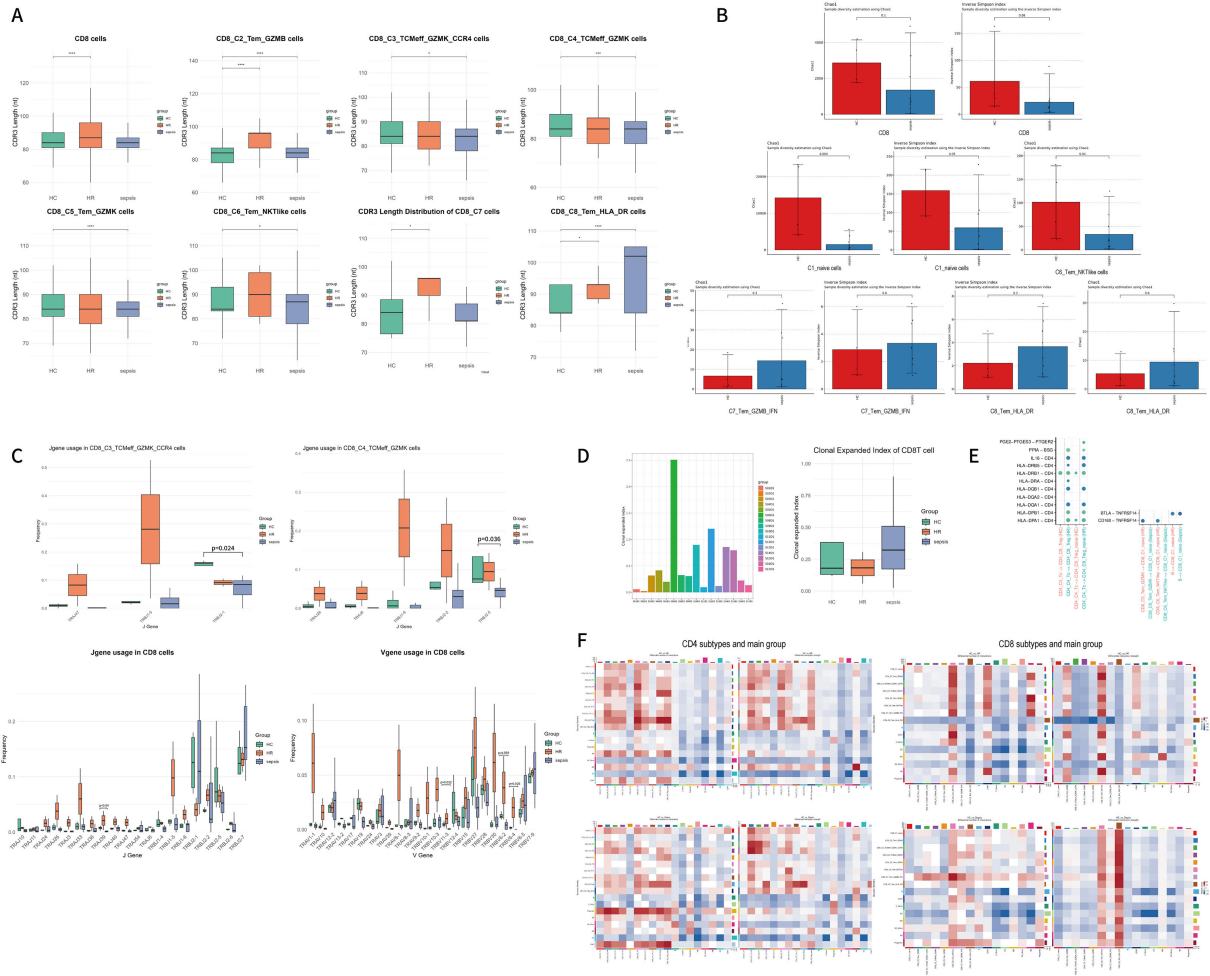
**(A)** nucleotide length distribution of CDR3 in total CD8^+^T cells and C2–8 subsets across HC, HR, and sepsis groups; **(B)** Bar plots showing the TCR repertoire diversity of total CD8^+^T cells and subtypes. Data were analyzed by an unpaired Mann-Whitney U-test; **(C)** Box plot showing the usage frequency of some TCR V and J genes across the three groups in designated subtypes. Statistical significance between groups was determined by unpaired Mann-Whitney U-test; **(D)** Comparison of clonal expansion index across groups and samples; **(E)** Dot plots showing ligand–receptor pairs (interaction) among cell subtypes across different patient groups; **(F)** Heatmaps of interactions between CD4^+^T and CD8^+^T cells and main cell groups. *p < 0.05, ***p < 0.001, and ****p < 0.0001.

We first evaluated the length distribution of CDR3 in TCR α and β chains across the overall CD8^+^ T cell population. The results showed that sepsis patients exhibited an increase in average CDR3 length compared to HCs, though this difference was not statistically significant. However, within multiple CD8^+^ T cell subsets from sepsis patients, CDR3 sequences were significantly longer than in HC, including C2_Tem_GZMB (mean difference = 1.32, t = -5.09, p < 0.001, effect size of Cohen d=0.19) and C8_Tem_HLA_DR, (mean difference = 9.63, t = -9.37, p < 0.001, effect size of Cohen d=1.10). In contrast, several other CD8^+^ T cell subsets showed significantly shortened CDR3 sequences in sepsis, including: C3_Tcm_eff_GZMK_CCR4 (mean difference = 1.25, t = 2.38, p = 0.018, effect size of Cohen d=0.15), C4_Tcm_eff_GZMK (mean difference = 1.98, t = 3.62, p < 0.001, effect size of Cohen d=0.26), C5_Tem_GZMK (mean difference = 2.16, t = 3.96, p < 0.001, effect size of Cohen d=0.29), C6_Tem_NKTlike (mean difference = 2.06, t = 2.49, p = 0.013, effect size of Cohen d=0.28) (**Figure 6A**).

HR subjects also displayed increased average CDR3 lengths in both the overall CD8^+^ T cell pool and specific subsets (C2_Tem_GZMB, C6_Tem_NKTlike, C7_Tem_GZMB_IFN, C8_Tem_HLA_DR). Notably, the overall CD8^+^ T cells (mean difference = 0.18, t = -6.73, p < 0.001, effect size of Cohen d=0.30), C2_Tem_GZMB (mean difference = 7.31, t = -14.65, p < 0.001, effect size of Cohen d=0.99), C7_Tem_GZMB_IFN (mean difference = 7.04, t = -2.48, p = 0.039, effect size of Cohen d=0.88), and C8_Tem_HLA_DR (mean difference = 5.84, t = -2.98,p = 0.023, effect size of Cohen d=1.18) subsets showed significant elongation compared to HC, suggesting that HR individuals exhibit CDR3 profile alterations similar to those in septic patients. No significant shortening of CDR3 sequences was observed in any subset among HR subjects (**Figure 6A**).

We further compared the gene expression profiles of CD8^+^ T cells between the sepsis and HC groups. Key findings in sepsis patients versus HC included: 1) Overall CD8^+^ T cells showed significantly reduced usage of the gene TRAJ39 (0.61%, 95%CI 0. 03%~1.01%, p=0.04), TRBV11-3 (0.39%, 95%CI 0.11%~0.73%, p=0.012), TRBV6-4 (0.17%, 95%CI 0.00%~0.29%, p=0.026), and TRBV6-2. 2 (-0.91%, 95%CI 0.19%~1.28%, p=0.039) The C3_Tcm_eff_GZMK_CCR4 subset exhibited decreased expression of the TRBJ2–1 gene (7.22%, 95%CI 2. 68%~12.29%, p=0.024). 3) The C4_Tcm_eff_GZMK subset demonstrated significantly reduced usage of the gene TRBJ2-5 (3.79%, 95%CI 0. 58%~23.39%, p=0.036) (**Figure 6C**).

##### 3.3.3.2 TCR repertoire diversity and clonality

We next compared the characteristics of TCR repertoire diversity among sepsis and HC individuals. Evaluation using both the inv.simp and the Chao1 index revealed that CD8^+^ T cells from sepsis group exhibited lower TCR diversity compared to HC. This suggests a reduction in the overall breadth of the peripheral T cell repertoire in sepsis patients—a finding consistent with the diversity patterns observed in CD4^+^ T cells.

In sepsis patients, reduced TCR diversity was observed across multiple CD8^+^ T cell subsets, including C1_naïve, C2_Tem_GZMB, C3_Tcm_eff_GZMK_CCR4, C4_Tcm_eff_GZMK, C5_Tem_GZMK, and C6_Tem_NKTlike. Among these, C1_naïve (inverse Simpson p = 0.05, Chao1 p = 0.004) and C6_Tem_NKTlike (Chao1 p = 0.04) subsets in sepsis patients showed significantly lower TCR diversity compared to HC (**Figure 6B**, **Supplementary Figure 2C**).

Sepsis patients exhibited clonal expansion in CD8^+^ T cell. Although sepsis patients showed the highest clonal expansion index compared to healthy controls (HC), this difference did not reach statistical significance (**Figure 6D**).

### 3.4 Dysregulated intracellular crosstalk within T cell subsets

Following global cell communication analysis, we observed that although the overall number and strength of interactions were reduced in both HR and sepsis groups compared to controls (HC), interactions among T, B, megakaryocyte (MK), and NK cells were increased. This prompted a focused investigation into T cell-centric communication dynamics.

#### 3.4.1 CD4^+^ T cell subset communication

The number and intensity of interactions among CD4^+^ T cell subsets increased in both HR and sepsis groups, primarily involving all subsets except C4_Tc. In the HR stage, cytotoxic CD4^+^ T cells (Tc) primarily acted as signal senders, exhibiting frequent interactions with Tregs. In contrast, during sepsis, Tc cells showed reduced both signal sending and receiving, which may be associated with the progressive activation of Tregs in early inflammation (**Figure 6F**). Compared to HC, significant alterations in HR-specific Tc→Treg interactions were identified in ligand-receptor pairs including PPIA-BSG, IL16-CD4, HLA-DPB1/HLA-DPA1/HLA-DQA1/HLA-DQB1/HLA-DRA/HLA-DRB1–CD4, CCL5-CCR4, and PEG2-PTGES3-PTGER2 (**Figure 6E**).

#### 3.4.2 CD8^+^ T cell subset communication

Similarly, communication activity among CD8^+^ T cell subsets increased in HR and sepsis patients, with the most pronounced changes occurring within the C6_Tem_NKTlike subset. During the HR stage, C6_Tem_NKTlike cells mainly functioned as signal receivers, whereas in sepsis, both C6_Tem_NKTlike and C8_Tem_HLA_DR emerged as major signal-accepting subsets (**Figure 6F**). Additionally, in sepsis, TNF signaling activation in C1_naïve CD8^+^ T cells appeared to be predominantly mediated by B cells via the BTLA–TNFRSF14 interaction axis (**Figure 6E**).

### 3.5 Protein-protein interaction network of the CD4^+^ C10_Tn_IFN subset and qPCR validation

**Figure 7:**
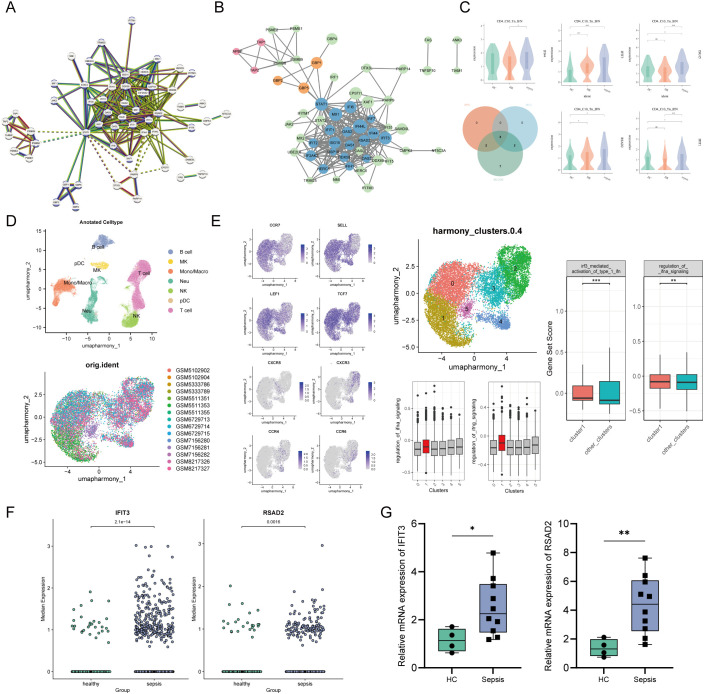
**(A)** Protein interaction network constructed based on 120 DGEs; **(B)** Protein interaction network plotted in Cytoscape software. Venn diagram of the intersection of hub genes identified by different methods; **(C)** Expression level comparisons of hub genes in C10_Tn_IFN; **(D)** UMAP visualization of 77,476 single cells generated from GEO dataset (GSE167363, GSE175453, GSE229173); **(E)** FeaturePlot visualization of marker gene expression across the UMAP embedding. UMAP of 13,481 CD4^+^T cells. Cluster 1 exhibited the highest enrichment score for interferon-related pathway genes (M982, M942), which was significantly elevated compared to all other subsets. **(F)** Expression level comparisons of hub genes *RSAD2, IFIT3* in CD4^+^Tn_IFN cells generated from GEO dataset. **(G)** qPCR comparisons of *RSAD2, IFIT3* in CD4^+^T cells generated from our cohort. Data were analyzed by an unpaired MannWhitney Utest, *p < 0.05, ****p < 0.01, and ***p < 0.001.

A total of 120 upregulated genes (log_2_FC ≥ 0.5) from the CD4^+^ C10_Tn_IFN subpopulation were used to construct a PPI network (confidence score > 0.9) (**Figure 7A**). Reactome pathway enrichment analysis identified 39 genes significantly associated with the interferon signaling pathway (HSA-913531). Key functional modules were subsequently identified using the MCODE, yielding three clusters: cluster 1 (19 nodes, 151 edges, MCODE score = 16.778), cluster 2 (3 nodes, 3 edges, MCODE score = 3), cluster 3 (3 nodes, 3 edges, MCODE score = 3) (**Figure 7B**). Using the cytoHubba plugin, eight hub genes related to the interferon pathway were identified: *IFI44, IFIT3, RSAD2, OAS2, ISG15, IFIT1, MX1*, and *OAS1* (**Figure 7C**).

Differential expression analysis revealed: *IFIT3* and *RSAD2* were upregulated in both sepsis and HR groups compared to HC. *ISG15* and *IFIT1* were significantly elevated in the sepsis group relative to HC. *IFI44* and *ISG15* showed higher expression in sepsis compared to the HR group (**Figure 7C**). To validate gene expression patterns, we reanalyzed scRNA-seq data from a publicly available GEO dataset. CD4^+^ T cell subpopulations were identified based on canonical marker expression. We identified a cell cluster in this dataset that exhibited the signature of C10_Tn_IFN observed in our study, characterized by high expression of *CCR7, SELL, LEF1*, and *TCF7*, along with significant enrichment of interferon-related pathways (**Figures 7D, E**). Within this CD4^+^ T subset, *IFIT3* and *RSAD2* were found to be significantly upregulated in sepsis patients compared to HC (**Figure 7F**). This result further supports the crucial role of these genes in regulating interferon signaling pathways in both septic and HR individuals. Therefore, we performed qPCR on peripheral blood CD4^+^ T cells from septic patients and controls collected in this study to validate the expression of these hub genes. The results confirmed that both *IFIT3* and *RSAD2* were significantly upregulated in sepsis compared to the control group (**Figure 7G**).

## 4 Discussion

Overall, by analyzing transcriptomic alterations in T lymphocytes from patients with sepsis, and individuals with active inflammation (HR), we observed that multiple CD4^+^ and CD8^+^ T cell subsets exhibited transcriptional signatures of increased activation and exhaustion during sepsis, alongside subset-specific modifications.

We identified two CD4^+^ T cell subsets, C7_Th1/17 and C10_Tn_IFN, which were significantly decreased in sepsis patients. These subsets exhibited enhanced activation of immune pathways and apoptotic characteristics in the HR group. Similarly, the C4_Tc subset demonstrated stronger cytotoxic activity in the high-risk state for urosepsis compared to the septic stage. The activation of the TCR pathway in Tc cells under sepsis combined with regression analyses, suggests their protective role in sepsis. However, it is important to note that the increased expression of cytolytic granules upon activation may contribute to the sustained inflammatory storm observed in septic patients (65, 66). These findings indicate that these CD4^+^ T cell subsets contribute to the pathophysiology in HR group, but the mechanism of their roles in mediating disease progression requires further exploration. For the newly defined C10_Tn_IFN subset, protein-protein interaction analysis suggested that *IFIT3* and *RSAD2* may serve as key regulatory targets for modulating interferon signaling pathways in this cell subpopulation. Both *IFIT3* and *RSAD2* are inflammation-related genes known for their protective roles in antiviral responses. Recent studies have also implicated them in cancer progression and chemotherapy sensitivity. For instance, IFIT3-activated T cells have been shown to recruit Tregs within the tumor microenvironment, thereby promoting immunosuppression (67–69). *RSAD2* has been recently found to mediate non-infectious cell necrosis. However, the role of interferon-activated T cells in bacterial-induced sepsis remains poorly characterized (70). Our qPCR validation confirms elevated transcriptional expression of the key genes *IFIT3* and *RSAD2* in bacterial sepsis, thereby providing preliminary evidence to address a gap in the current understanding of this field. It should be noted that protein−level validation was not performed in this study, which represents an important direction for future research. In contrast, within Treg cell subsets, although they receive signals from CD4^+^ Tc cells in patients at high risk, their immunosuppressive functions only become gradually activated in sepsis, exacerbating immune and inflammatory dysregulation (39, 71).

In sepsis, we observed a broad increase in CD8^+^ effector memory subsets. Among these, only C7_Tem_GZMB_IFN showed a decreasing trend in septic groups. The CD8_C1_naïve subset displayed differential enrichment in IFN and TNF pathways across pathological conditions. Previous studies suggest that IFN primarily activates immune responses promoting cytolytic T cell formation, whereas prolonged stimulation may induce exhaustion and inhibitory feedback (72–74). TNF, a pleiotropic pro-inflammatory cytokine, is central to CTL activation, differentiation, and cytotoxicity (75). Additionally, we identified a transitional cell population between Tcm and Tem, termed Tcm_eff, characterized by anti-apoptotic features and weak effector function compared to Tem. Although common viral infections were clinically excluded, observed phenotypic shifts in CD8^+^ T cells underscore their significant role in bacterial sepsis (76).

Alterations in VJ gene usage and CDR3 sequence length within CD4^+^ and CD8^+^ T cell subsets in sepsis suggest that the septic microenvironment drives modifications in the encoding of TCR CDR3 sequences (56). This highlights T cell developmental changes under sepsis conditions (57, 77), extending previous studies focused on bulk T cells (39, 65, 66, 71). It is noteworthy that CDR3 sequences are analyzed at the single−cell level, the large number of cells may yield statistically significant differences that are not necessarily large in effect size, as indicated by Cohen’s d analysis. Given the limitations of transcriptomic sequencing, a deeper understanding of CDR3 dynamics under disease conditions will require complementary validation at the protein level of the TCR. Divergent CDR3 length dynamics across CD8^+^ subsets may reflect distinct TCR recovery mechanisms post-sepsis, where CD8^+^ T cells proliferate and gradually reconstitute the TCR repertoire regardless of clonal deletion (78–81). Compared to healthy individuals, the TCR repertoires of most CD4^+^ and CD8^+^ T cell subsets in septic patients did not show statistically significant differences. This indicates that, although overall TCR diversity is reduced in sepsis, certain T cell subsets remain relatively unperturbed, potentially playing critical roles in disease progression.

Urinary tract stones, a common clinical condition, can progress to urosepsis regardless of surgical intervention (82). Predictive models based on clinical data have identified several risk factors for urosepsis, yet the immune mechanisms underlying high-risk populations remain poorly described (7–10). This study elucidates transcriptional and phenotypic changes in T cell subsets among septic and HR patients, revealing distinct CD4^+^ and CD8^+^ subpopulations and their potential mechanisms influencing disease states. These insights provide a reference for future validation of functional alterations in T−cell subsets in sepsis−high−risk populations, and by identifying key genes associated with bacterial sepsis, offer a perspective for immunologically based prevention and monitoring of urosepsis in patients with urinary stones.

This study included only a limited number of HR patients, and further expansion of the sample size is required to more comprehensively characterize the immune profiles of this population. Additionally, the heterogeneity of pathogens infection sites triggering sepsis in our cohort may introduce confounding factors in the analysis of TCR repertoire and transcriptomic changes. Future studies should aim to enroll larger cohorts of patients with well−defined pathogen types and specific infection sources to obtain more precise characterizations of CDR3 dynamics and T−cell immune signatures.

## Data Availability

The datasets presented in this study can be found in online repositories. The names of the repository/repositories and accession number(s) can be found below: https://ngdc.cncb.ac.cn/gsa-human, GSA-Human: HRA013466. (i.e., refs. 83 and 84 in the current manuscript). Local law prohibits depositing raw datasets derived from human samples outside of the country of origin. You can request access through https://bigd.big.ac.cn/gsub/.

## Supplementary material

Supplementary Figure 1 **(A)** Heatmap showing the overall signaling patterns in HR vs. HC and sepsis vs. HC. Circle plots demonstrate an overall trend of decreased number and strength of interactions in sepsis compared with HC; **(B)** FeaturePlot visualization of marker gene expression across the UMAP of CD4^+^T embedding; **(C)** Enrichment analysis (GO terms and KEGG pathways) of upregulated genes in CD4^+^T subsets from sepsis/HR compared to HC subjects; **(D)** Bar plot showing the TCR repertoire diversity of CD4^+^T cell subsets.

Supplementary Figure 2 **(A)** Heatmap of average expression of marker genes in each labeled cell type; **(B)** Enrichment analysis (GO terms and KEGG pathways) of upregulated genes in CD8^+^T subsets from sepsis/HR compared to HC subjects; **(C)** Bar plot showing the TCR repertoire diversity of CD8^+^T cell subsets.
