# Supplementary material for: Distinct landscapes of T-cell immunity and TCR repertoire between sepsis and pre-septic high-risk states
Source: Front Immunol. 2026 Mar 3;17:1754842. doi: 10.3389/fimmu.2026.1754842 (PMC12992028; doi:10.3389/fimmu.2026.1754842)
Supplement: Supplementary file 3 [file Table3.docx]

Table 3. Clinical Characteristics and Cytokine Levels in Sepsis Group vs Control Group. Continuous variables: median (Q1–Q3). Mann-Whitney U test used for between-group comparisons.

| **Variable** | **Sepsis (N=10)** | **Control (N=5)** | **p-value** |
| --- | --- | --- | --- |
| Age (years) | 71.5 (65.2–83.5) | 72 (67–73) | 0.75900 |
| Temperature (℃) | 37.7 (36.5–38.9) | 36.4 (36.3–36.4) | 0.15600 |
| Respiratory rate (/min) | 22 (18.8–24.5) | 17.5 (17.2–17.8) | 0.15900 |
| Heart rate (/min) | 100.5 (88.8–105.5) | 76 (73–79) | 0.10700 |
| WBC (×10⁹/L) | 11.6 (9.5–13.6) | 5.8 (4.7–8.1) | 0.07580 |
| Neutrophil (×10⁹/L) | 9.6 (6.7–12) | 3.6 (2.5–5.6) | 0.01690 |
| Lymphocyte (×10⁹/L) | 0.5 (0.2–1.1) | 2 (1.8–2.2) | 0.04310 |
| Hb (g/L) | 110 (89–139.5) | 131 (130–133) | 0.19800 |
| PLT (×10⁹/L) | 153 (91.2–232.2) | 170 (126–254) | 0.58200 |
| CRP (mg/L) | 126 (108–158) | 4.1 (2.6–5.6) | 0.04510 |
| PCT (ng/mL) | 34.8 (3.4–49.4) | 0.1 (0.1–0.1) | 0.04090 |
| Albumin (g/L) | 31.4 (27.7–33) | 38.6 (37.7–41.2) | 0.00846 |
| ALT (U/L) | 39.8 (18.4–54.9) | 16.3 (11.6–23.7) | 0.14100 |
| AST (U/L) | 35.1 (28–135.8) | 15.4 (14.7–16.9) | 0.01690 |
| Creatinine (μmol/L) | 119.7 (85.5–202.6) | 109.8 (63.3–115.1) | 0.42600 |
| D-Dimer (mg/L) | 2 (1.5–4.5) | 0.1 (0.1–0.2) | 0.00267 |
| IL-1β | 2.58 (0.47–5) | 8.47 (2.13–8.76) | 0.51900 |
| IL-2 | 1.33 (0.84–8.47) | 2.68 (1.94–2.81) | 0.92700 |
| IL-4 | 0.84 (0.43–1.54) | 1.66 (1.42–3.54) | 0.26200 |
| IL-5 | 1.79 (0.79–9) | 7.67 (1.68–8.54) | 0.92700 |
| IL-6 | 295.53 (97.08–3269.26) | 4.54 (4.47–4.65) | 0.00577 |
| IL-8 | 314.91 (57.83–553.5) | 55.4 (17.95–80.17) | 0.64800 |
| IL-10 | 21.59 (8.63–56.97) | 1.24 (1.23–1.36) | 0.02250 |
| IL-12p70 | 1.04 (0.7–2.05) | 1.36 (1.26–1.76) | 0.92700 |
| IL-17 | 19.73 (4.31–58.16) | 4.01 (2.08–4.87) | 0.27200 |
| TNF-α | 1.09 (0.67–1.51) | 2.76 (2.22–3.43) | 0.26100 |
| IFN-γ | 30.66 (8.69–49.29) | 3.54 (1.76–3.93) | 0.31100 |
| IFN-α | 0.83 (0.58–1.05) | 2.78 (1.24–2.97) | 0.20000 |
