# Supplementary material for: Distinct landscapes of T-cell immunity and TCR repertoire between sepsis and pre-septic high-risk states
Source: Front Immunol. 2026 Mar 3;17:1754842. doi: 10.3389/fimmu.2026.1754842 (PMC12992028; doi:10.3389/fimmu.2026.1754842)
Supplement: Supplementary file 4 [file Table4.docx]

Table 4. Clinical Characteristics and Cytokine Levels in High-risk Group vs Control Group. Continuous variables presented as median (Q1–Q3). Between-group comparisons performed using Mann-Whitney U test.

| **Variable** | **High-risk (N=6)** | **Control (N=5)** | **p-value** |
| --- | --- | --- | --- |
| Age (years) | 71.5 (64.5–77.8) | 72 (67–73) | 0.8550 |
| Temperature (℃) | 37.8 (36.8–38.5) | 36.4 (36.3–36.4) | 0.1770 |
| Respiratory rate (/min) | 20 (18.5–21.5) | 17.5 (17.2–17.8) | 0.2350 |
| Heart rate (/min) | 71.5 (70–95.5) | 76 (73–79) | 1.0000 |
| WBC (×10⁹/L) | 8.8 (6.3–15.4) | 5.8 (4.7–8.1) | 0.1710 |
| Neutrophil (×10⁹/L) | 7.8 (6–14.3) | 3.6 (2.5–5.6) | 0.0552 |
| Lymphocyte (×10⁹/L) | 1 (0.3–2) | 2 (1.8–2.2) | 0.2350 |
| Hb (g/L) | 113 (109.8–118.5) | 131 (130–133) | 0.0358 |
| PLT (×10⁹/L) | 144.5 (109.2–208.2) | 170 (126–254) | 0.5230 |
| CRP (mg/L) | 95.5 (36–141.3) | 4.1 (2.6–5.6) | 0.0668 |
| PCT (ng/mL) | 2.3 (0.6–23.4) | 0.1 (0.1–0.1) | 0.0651 |
| Albumin (g/L) | 31 (27.7–35.6) | 38.6 (37.7–41.2) | 0.0828 |
| ALT (U/L) | 13.4 (12.6–14.5) | 16.3 (11.6–23.7) | 0.6480 |
| AST (U/L) | 19.4 (18.9–21.4) | 15.4 (14.7–16.9) | 0.2000 |
| Creatinine (μmol/L) | 77.7 (53.5–103) | 109.8 (63.3–115.1) | 0.1710 |
| D-Dimer (mg/L) | 1.8 (1.2–2.4) | 0.1 (0.1–0.2) | 0.0786 |
| IL-1β | 2.25 (2.25–2.27) | 8.47 (2.13–8.76) | 0.9160 |
| IL-2 | 2.39 (1.11–2.81) | 2.68 (1.94–2.81) | 0.9170 |
| IL-4 | 1.58 (0.43–1.64) | 1.66 (1.42–3.54) | 0.3980 |
| IL-5 | 2.43 (0.58–3.08) | 7.67 (1.68–8.54) | 0.6720 |
| IL-6 | 48 (36.34–685.9) | 4.54 (4.47–4.65) | 0.0122 |
| IL-8 | 26.81 (6.87–116.04) | 55.4 (17.95–80.17) | 0.9170 |
| IL-10 | 10.81 (1.89–23.71) | 1.24 (1.23–1.36) | 0.0947 |
| IL-12p70 | 1.67 (1.59–1.77) | 1.36 (1.26–1.76) | 0.6760 |
| IL-17 | 10.36 (7.79–10.67) | 4.01 (2.08–4.87) | 0.0601 |
| TNF-α | 1.95 (0.67–1.96) | 2.76 (2.22–3.43) | 0.0907 |
| IFN-γ | 30.67 (2.71–31.54) | 3.54 (1.76–3.93) | 0.4030 |
| IFN-α | 1.51 (1.25–1.82) | 2.78 (1.24–2.97) | 0.4630 |
