# Supplementary material for: Distinct landscapes of T-cell immunity and TCR repertoire between sepsis and pre-septic high-risk states
Source: Front Immunol. 2026 Mar 3;17:1754842. doi: 10.3389/fimmu.2026.1754842 (PMC12992028; doi:10.3389/fimmu.2026.1754842)
Supplement: Supplementary file 5 [file Table5.docx]

Table 5. Univariate and multivariate logistic regression analysis of cell cluster signatures associated with sepsis diagnosis.

| **Variable** | **Healthy control (N=43)** | **Sepsis (N=116)** | **Univariate** **logistic regression, OR (95%CI)** | **Multivariate logistic regression, OR (95%CI)** |
| --- | --- | --- | --- | --- |
| C1_Tn | -23.3 ± 13.9 | -55.1 ± 22.6 | 0.93 (0.91-0.95, p<.001) | 0.88 (0.76-1.01, p=.062) |
| C2_Th1 | -59.1 ± 9.6 | -88.1 ± 17.5 | 0.85 (0.81-0.90, p<.001) | 0.95 (0.84-1.08, p=.428) |
| C3_Th2 | 103.2 ± 35.8 | 119.3 ± 96.9 | 1.00 (1.00-1.01, p=.289) | 0.98 (0.96-1.01, p=.205) |
| C4_Tc | 340.8 ± 211.1 | -25.4 ± 137.3 | 0.99 (0.98-0.99, p<.001) | 0.97 (0.94-0.99, p=.003) |
| C5_Tfh | 0.4 ± 2.5 | -1.2 ± 3.3 | 0.86 (0.77-0.96, p=.007) | 2.05 (1.11-3.78, p=.021) |
| C6_Th17 | 118.9 ± 70.1 | 104.3 ± 112.8 | 1.00 (1.00-1.00, p=.425) | 0.89 (0.82-0.97, p=.008) |
| C7_Th1_17 | 185.9 ± 151.7 | 115.4 ± 206.8 | 1.00 (1.00-1.00, p=.046) | 1.05 (1.01-1.09, p=.013) |
| C8_Treg | -23.1 ± 17.7 | -51.8 ± 24.2 | 0.95 (0.93-0.97, p<.001) | 1.07 (1.00-1.13, p=.038) |
| C9_Treg_naive | 3.3 ± 3.5 | -1.7 ± 3.7 | 0.71 (0.63-0.80, p<.001) | 0.44 (0.25-0.80, p=.007) |
| C10_Tn_IFN | -119.2 ± 25.7 | -162.6 ± 72.7 | 0.99 (0.98-1.00, p<.001) | 1.00 (0.97-1.03, p=.929) |
| age | 63.2 ± 10.5 | 63.5 ± 15.0 | 1.00 (0.98-1.03, p=.900) | 1.00 (0.90-1.11, p=.946) |
| gender | 16 (37.2%) | 38 (32.8%) |  |  |
|  | 27 (62.8%) | 78 (67.2%) | 1.22 (0.59-2.52, p=.599) | 2.09 (0.26-17.04, p=.489) |
