# Supplementary figures and images for: Distinct landscapes of T-cell immunity and TCR repertoire between sepsis and pre-septic high-risk states
Source: Front Immunol. 2026 Mar 3;17:1754842. doi: 10.3389/fimmu.2026.1754842 (PMC12992028; doi:10.3389/fimmu.2026.1754842)

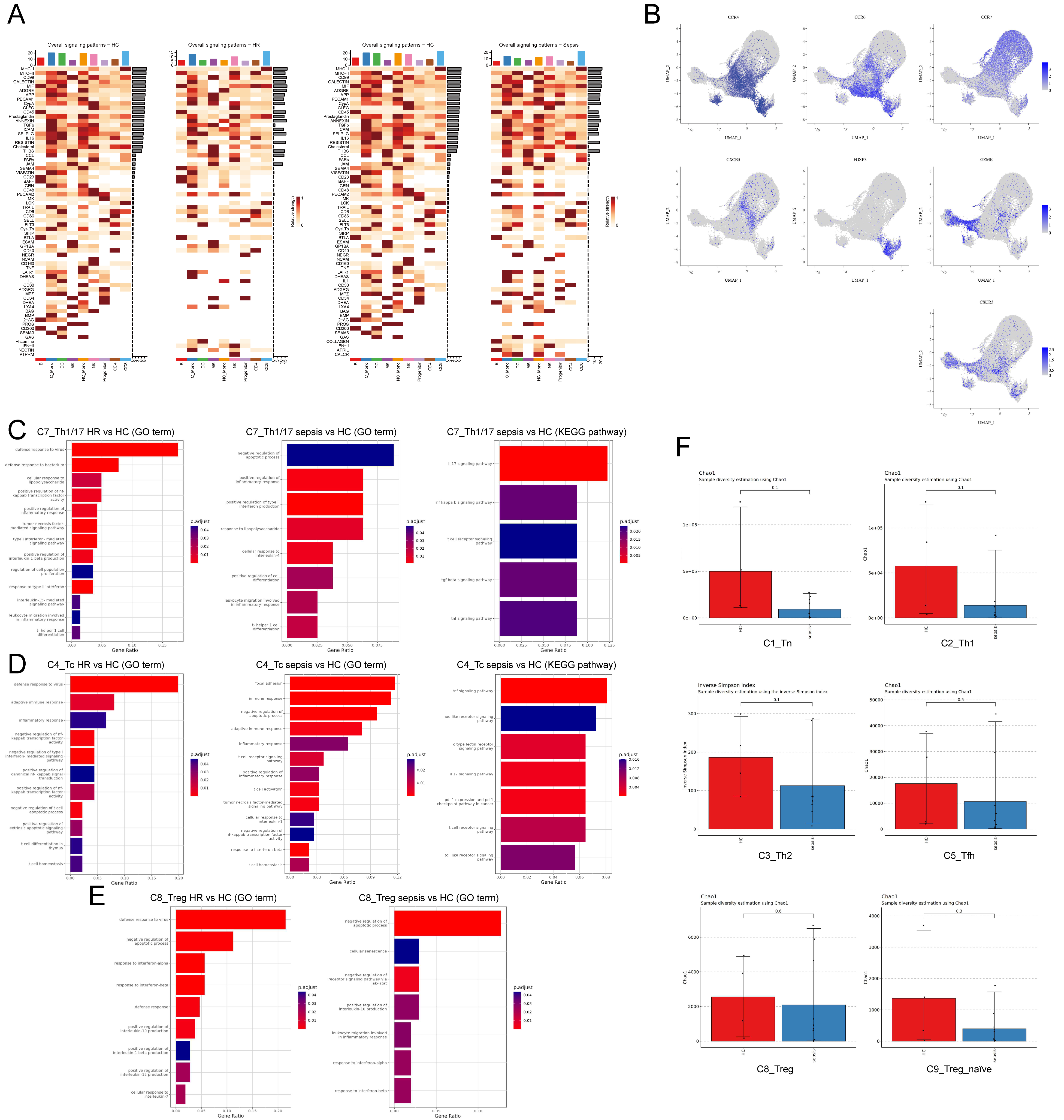

Supplement: Supplementary Figure 1 — (A) Heatmap showing the overall signaling patterns in HR vs. HC and sepsis vs. HC. Circle plots demonstrate an overall trend of decreased number and strength of interactions in sepsis compared with HC; (B) FeaturePlot visualization of marker gene expression across the UMAP of CD4+T embedding; (C) Enrichment analysis (GO terms and KEGG pathways) of upregulated genes in CD4+T subsets from sepsis/HR compared to HC subjects; (D) Bar plot showing the TCR repertoire diversity of CD4+T cell subsets. [file Image1.tif]

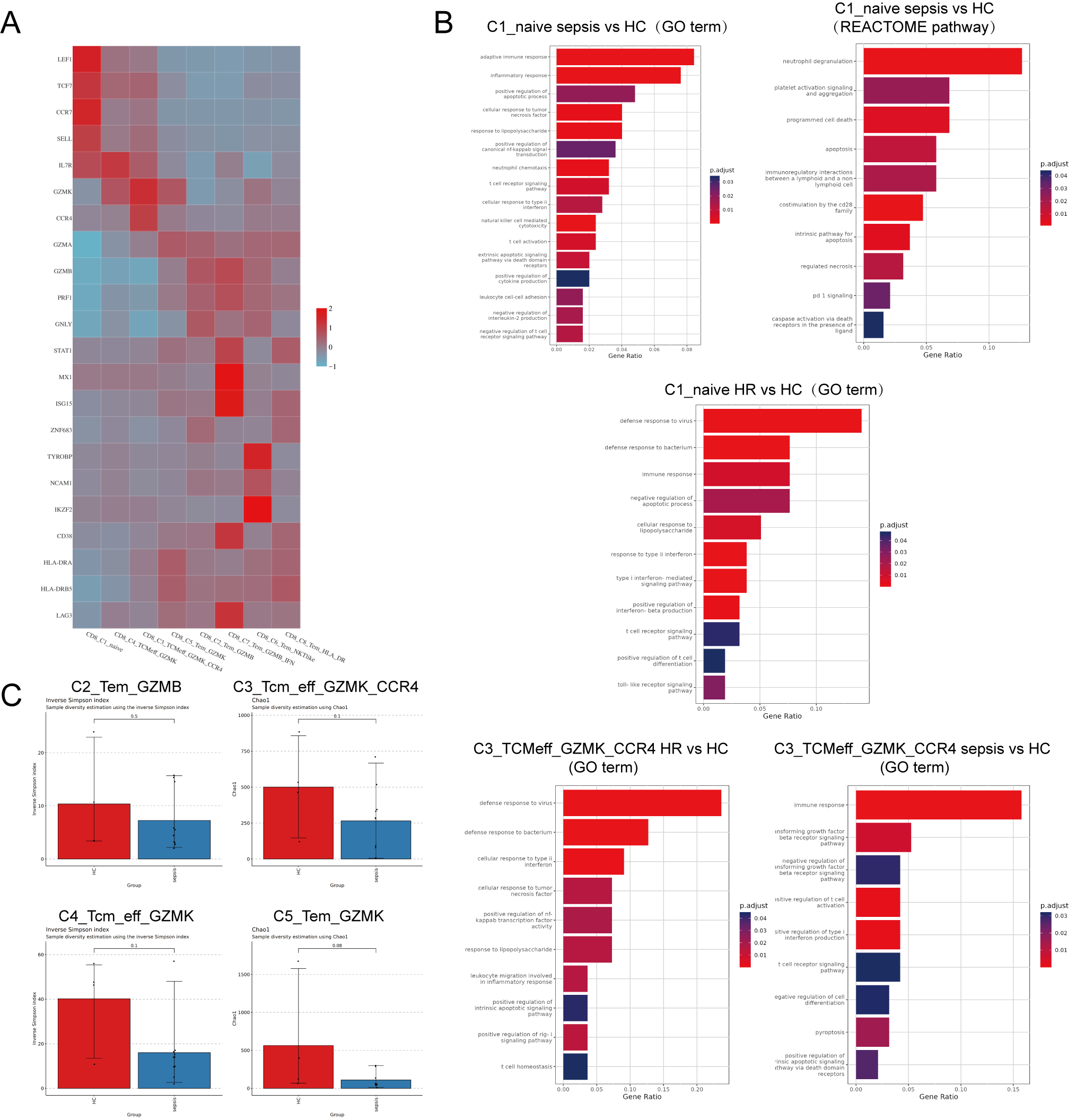

Supplement: Supplementary Figure 2 — (A) Heatmap of average expression of marker genes in each labeled cell type; (B) Enrichment analysis (GO terms and KEGG pathways) of upregulated genes in CD8+T subsets from sepsis/HR compared to HC subjects; (C) Bar plot showing the TCR repertoire diversity of CD8+T cell subsets. [file Image2.tif]
